# Potent inhibitors of toxic alpha-synuclein identified via cellular time-resolved FRET biosensors

**DOI:** 10.1038/s41531-021-00195-6

**Published:** 2021-06-28

**Authors:** Anthony R. Braun, Elly E. Liao, Mian Horvath, Prakriti Kalra, Karen Acosta, Malaney C. Young, Noah Nathan Kochen, Chih Hung Lo, Roland Brown, Michael D. Evans, William C. K. Pomerantz, Elizabeth Rhoades, Kelvin Luk, Razvan L. Cornea, David D. Thomas, Jonathan N. Sachs

**Affiliations:** 1 Department of Biomedical Engineering, University of Minnesota, Minneapolis, MN USA; 2 Department of Pathology and Laboratory Medicine, University of Pennsylvania, Philadelphia, PA USA; 3 Department of Chemistry, University of Minnesota, Minneapolis, MN USA; 4 Biochemistry & Molecular Biophysics Graduate Group, University of Pennsylvania, Philadelphia, PA USA; 5 Clinical and Translational Science Institute, University of Minnesota, Minneapolis, MN USA; 6 Department of Chemistry, University of Pennsylvania, Philadelphia, PA USA; 7 Department of Biochemistry, Molecular Biology and Biophysics, University of Minnesota, Minneapolis, MN USA; 8 Photonic Pharma LLC, Minneapolis, MN USA

**Keywords:** Parkinson's disease, High-throughput screening

## Abstract

We have developed a high-throughput drug discovery platform, measuring fluorescence resonance energy transfer (FRET) with fluorescent alpha-synuclein (αSN) biosensors, to detect spontaneous pre-fibrillar oligomers in living cells. Our two αSN FRET biosensors provide complementary insight into αSN oligomerization and conformation in order to improve the success of drug discovery campaigns for the treatment of Parkinson’s disease. We measure FRET by fluorescence lifetime, rather than traditional fluorescence intensity, providing a structural readout with greater resolution and precision. This facilitates identification of compounds that cause subtle but significant conformational changes in the ensemble of oligomeric states that are easily missed using intensity-based FRET. We screened a 1280-compound small-molecule library and identified 21 compounds that changed the lifetime by >5 SD. Two of these compounds have nanomolar potency in protecting SH-SY5Y cells from αSN-induced death, providing a nearly tenfold improvement over known inhibitors. We tested the efficacy of several compounds in a primary mouse neuron assay of αSN pathology (phosphorylation of mouse αSN pre-formed fibrils) and show rescue of pathology for two of them. These hits were further characterized with biophysical and biochemical assays to explore potential mechanisms of action. In vitro αSN oligomerization, single-molecule FRET, and protein-observed fluorine NMR experiments demonstrate that these compounds modulate αSN oligomers but not monomers. Subsequent aggregation assays further show that these compounds also deter or block αSN fibril assembly.

## Introduction

Preventing or reversing the pathological misfolding and self-association of α-synuclein (αSN) can rescue a broad spectrum of pathological cellular insults that manifest in Parkinson’s disease (PD), dementia with Lewy bodies, multiple systems atrophy, and other alpha-synucleinopathies^1–9^. Over the past several years, the field’s understanding of which forms of αSN assemblies are most toxic has evolved, shifting away from mature fibrils rich in β-sheets toward structurally heterogeneous, early-stage oligomers^9–14^. Many questions remain regarding these heterogeneous oligomers, including what their exact macromolecular constituency is in cells (i.e., what co-mingles with αSN^1,15–17^), whether they possess any well-defined structural motifs—various forms, including a tetramer, have been described in the literature^18–24^—and how variations in their molecular properties contribute to toxicity. These unknowns complicate drug discovery, where it remains to be seen whether a unique small-molecule binding site or epitope even exists, as has been suggested recently for fibrils^25–27^. More generally, it is unclear whether direct binding of an inhibitor to αSN oligomers, or fibrils for that matter, is more efficient than indirectly targeting the cellular machinery and pathways that can prevent or alter their assembly^28^.

Despite many unknowns, there is clear therapeutic potential in shifting the equilibrium distribution of αSN monomers, oligomers, and fibrils^4,26,27,29–36^. Unfortunately, it has proven experimentally challenging to either control the stochasticity and heterogeneity of in vitro oligomerization of purified αSN or to directly monitor pre-fibrillar oligomers in cells. Consequently, most discovery campaigns have targeted in vitro fibril growth. A recent study that used thioflavin-T (ThT) as the engine for a high-throughput screening (HTS) campaign identified an inhibitor of purified αSN fibrillization that reduced inclusions in human neuroglioma (H4) cells^27^. Nevertheless, even at 10 μM, the inhibitor was only effective in 50% of cells^27^. The same inhibitor also protected neuron loss in a *Caenorhabditis elegans* model of PD but again in less than half the worms^27^. Another recent HTS study used Förster or fluorescence resonance energy transfer (FRET) to monitor sodium dodecyl sulfoxide (SDS)-induced fibrillization of recombinant αSN^37^. Hits identified in that study predominantly targeted monomeric αSN (αSN(m)), with the best hit compounds rescuing oligodendrocytes from exogenously added αSN aggregate-induced cell death, albeit at high compound concentrations (30 μM)^37^. While targeting αSN fibrils has shown promise, concerns persist as to whether these inhibitors of purified αSN fibrils translate to the more complex assemblies (e.g., non-fibrillar oligomers) formed in cells and if they can be cytoprotective in the low nanomolar to picomolar range (as antibodies are^38–40^).

Anle138b is a particularly prominent example of a small-molecule inhibitor of αSN oligomerization (with an apparent EC_50_ of 2.8 μM). Anle138b prevents the formation of iron-induced αSN aggregates in vitro and shows strong positive effects in mouse models of PD^41^. In our studies below, we show that Anle138b protects SH-SY5Y cells from αSN-induced death with an EC_50_ of ≈900 nM. A more recent study reached nanomolar potency (500 nM) using engineered peptides that mimic fibril structures and prevented the growth of pre-formed fibril (PFF) seeds with a single 25 μM inhibitor dose required for cytoprotection^25^.

There is one recent study that has resulted in small-molecule inhibitors of non-fibrillar aggregates. In a technological feat, bimolecular fluorescence complementation (BiFC) was used to monitor αSN oligomerization in a cellular HTS campaign, yielding small molecules that protected H4 cells in the mid-nanomolar range (reported IC_50_ ≈ 500 nM)^42^. Here we adopt a variation on this same theme by engineering FRET-based biosensors that monitor αSN oligomers in cells without requiring direct reporter protein interaction and maturation of the fluorophore, which is required in BiFC. Our αSN FRET cellular biosensor screening platforms are engineered to target toxic, early-stage, spontaneous αSN oligomers and conformations. Using single mEGFP and TagRFP fusion constructs (inter-protomeric FRET) and double-fusion constructs (intra-protomeric FRET), these two biosensors monitor oligomerization and conformation by using fluorescence lifetimes (FLTs). A key advantage of our approach is the detection of FLTs rather than the traditional fluorescence intensities. This increases the precision of the measurements by a factor of thirty^43^, enabling detection of minute structural changes within the ensemble of αSN assemblies and thereby the discovery of small molecules that may otherwise be missed with traditional measurements.

The inter-protomeric and double-fusion (intra-protomeric) FRET biosensors were transiently transfected into HEK293 cells and HTS were conducted on the 1280-compound Library of Pharmacologically Active Compounds (LOPAC) to validate our screening platforms. Together, these two biosensors provide complementary insight into αSN oligomerization and conformation. We then tested the efficacy of hit compounds in SH-SY5Y cells overexpressing unlabeled wild-type (WT) αSN (to rule out labeling artifacts), and we report that two small molecules, Demeclocycline HCl (DEM) and Ro 90–7501 (RO), completely protected SH-SY5Y cells from αSN-induced death. The compounds are active in the low nanomolar range (EC_50_ = 65 and 78 nM, respectively) and are an order of magnitude more potent than previously described small molecules. We tested the efficacy of these compounds in a primary mouse neuron assay of αSN pathology (phosphorylation of mouse αSN PFFs) and show rescue for DEM (but not RO), as well as another lead compound ((±)-Bay K 8644, BAY) that was not effective in the SH-SY5Y cell models. Given the very different nature of this murine model from the screening platform, it is notable to have found effective compounds.

We further characterized these three hits with a combination of biochemical and biophysical assays to determine potential mechanism of action (MOA) for the rescue of αSN-induced pathology. Using recombinant protein oligomerization and fibrillization assays coupled to single-molecular FRET (smFRET) and protein-observed fluorine (PrOF) nuclear magnetic resonance (NMR) experiments, we confirmed that these compounds directly interact with αSN oligomers, increasing the extent of oligomerization and shifting the equilibrium toward non-toxic, off-pathway αSN oligomers. Subsequent seeded ThT assays demonstrate that these compounds also deter or block fibril assembly (at a sub-stoichiometric ratio to αSN(m)), further demonstrating the complex interplay between pre-fibrillar oligomers, beta-sheet fibrils, and cell death.

Our inter- and intra-protomeric FRET αSN biosensors provide a new platform for evaluating αSN conformation and oligomerization. Our HTS screens using these FRET αSN biosensors identified new hit compounds that target αSN pathology at nanomolar potencies and validated multiple compounds that were identified in previous screens using other HTS platforms. We further demonstrate the ability of our drug-discovery platform to identify hit compounds that can target a multitude of therapeutic MOAs, which further demonstrates the broad potential application of our cellular αSN oligomer FRET biosensors.

## Results

### αSN biosensor engineering

The cellular αSN biosensors developed here monitor spontaneous oligomerization and the ensemble of conformations of αSN (Supplementary Fig. 1). Each αSN construct is fused with either donor (N-terminal mEGFP) or acceptor (C-terminal TagRFP) or both (mEGFP-αSN-TagRFP), where all XFPs are monomerized forms^44^. The HTS strategy employed here uses HEK293 cells and a fluorescence lifetime plate reader (FLT-PR) platform designed to monitor specific protein interactions and structural changes in living cells^43,45,46^. FLT detection increases the precision of FRET-based screening by a factor of 30 compared with conventional fluorescence intensity detection^43^ and provides exquisite sensitivity to resolve minute structural changes within protein ensembles. The improved signal to noise is due to FLT being an intrinsic property of the XFP and not sensitive to fluctuations in steady-state fluorescence intensity. This sensitivity, along with our series of αSN–XFP fusion constructs, allows for the monitoring of both inter-protomeric αSN interactions and oligomeric conformations.

**Fig. 1:**
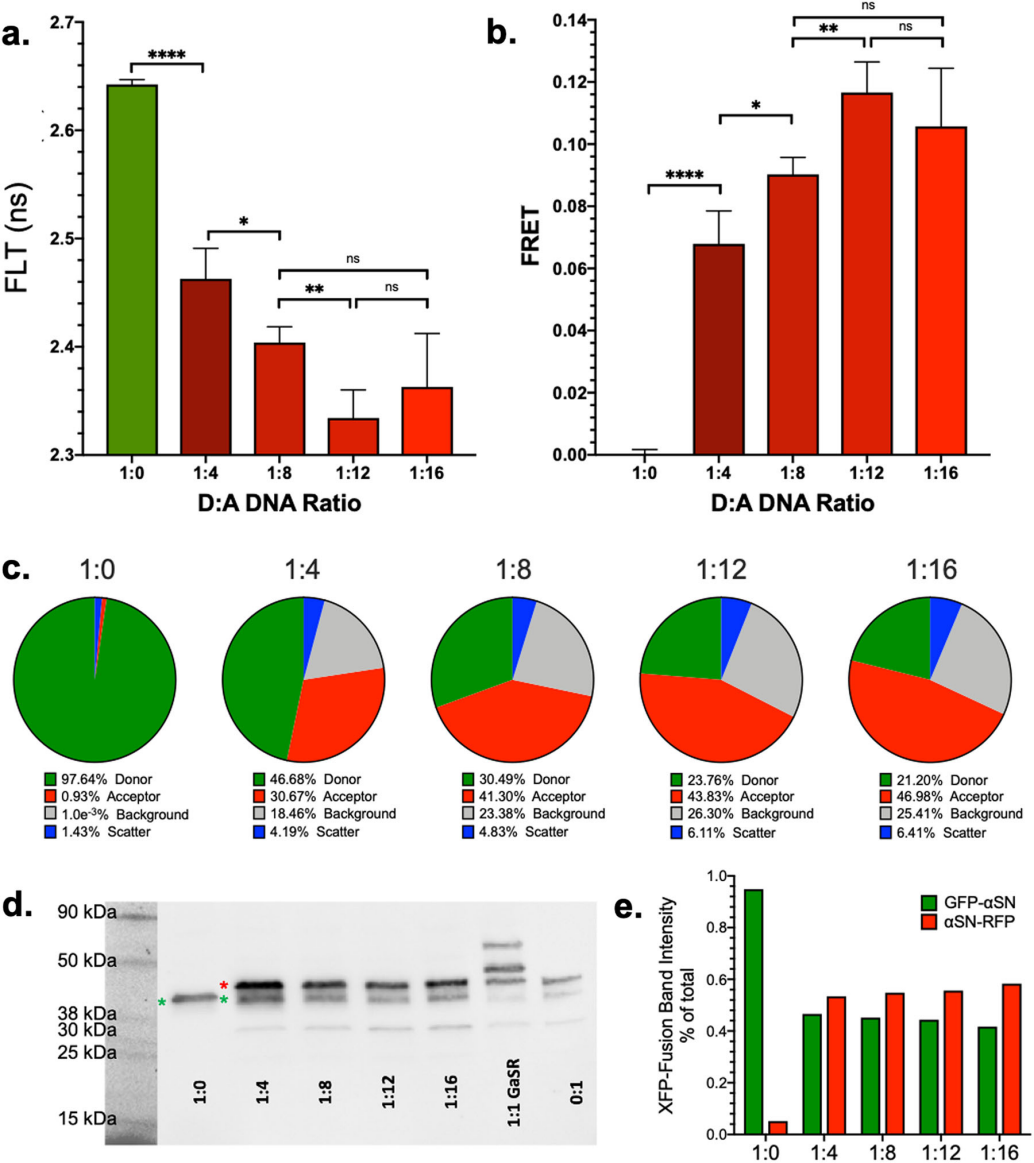
Engineering of αSN cellular FRET biosensors. **a** Fluorescence lifetime (FLT) measured with the FLT-PR for a range of donor and acceptor (D:A) ratios show significant reduction of FLT that saturates around D:A of 1:8. **b** FRET determined at each D:A ratio in **a**. **c** Spectral unmixing of each biosensor illustrates the shift in ratio of D:A throughout the increasing D:A DNA titration (see Supplementary Fig. 3 for source spectra). The relative donor-to-background signal (gray to green) is directly correlated to the degree of variance for each system. **d** Immunoblot for αSN in WT αSN oligomer (inter-protomeric) biosensor and intra-protomeric biosensor. Cell lysates for each D:A ratio were analyzed via SDS-PAGE gel and probed for αSN using the Syn1 antibody. All samples were transfected with a constant total DNA (15 μg DNA for 10 cm plate). GFP-αSN manifests as a strong band at ~40 kD in the 1:0 lane. The C-terminal RFP-αSN fusion runs slightly higher than the GFP-αSN construct. Similarly, C-terminal truncation is observed for αSN-RFP. Note: RFP truncation does not negatively affect our FRET assay as we only monitor the FLT for the donor (GFP construct). Supplementary Fig. 6 explores αSN truncation in more depth via comparisons of total αSN (Syn1) and LB509-positive immunoblots. **e** Densitometric analysis of the two most prominent XFP bands (indicated by green and red asterisks (*) in **d** shows an increase in acceptor expression, albeit at a reduced rate relative to D:A DNA titration. Experiments were performed with *n* = 3; *p < 0.05, **p < 0.01, ****p < 0.0001, ns indicates not significant as determined by Student’s *t* test.

Optimization of expression conditions of FLT biosensor was determined by transiently transfecting a titration of donor-to-acceptor (D:A) ratios into HEK293 cells. Cells were harvested after 48 h of transfection and then plated and read in the FLT-PR. Figure 1a presents the FLT of each condition with the corresponding FRET in Fig. 1b. We observe a decrease in FLT (i.e., increased FRET) up to a plateau that transitions at D:A of 1:8. The magnitudes of the measured lifetimes (and hence FRET, Figs. 1a, b and 3) are slightly higher than observed for our recent tau biosensor^47^ and roughly consistent with other biosensors using this FLT platform^43,45–52^. Figure 3 shows the results from the intra-protomeric, double-fusion biosensor, which for WT αSN had a slightly lower, though still substantial, FRET signal.

We next characterized the relative fluorescence contribution of donor and acceptor αSN constructs using a spectral unmixing plate reader (SUPR), which records the entire emission spectra. Subsequent data analysis resolves the contributions from the four primary spectral components: (1) donor fluorescence, (2) acceptor fluorescence, (3) cellular autofluorescence background, and (4) Raman scattering. Supplementary Fig. 2 highlights control spectra and Supplementary Fig. 3 presents the spectra from the titration in Fig. 1a. Quantification of the spectral unmixing fits is presented in Fig. 1c. As expected, we observe a clear increase in the acceptor fluorescence relative to the donor at higher DNA titrations.

The SUPR experiment provides further guidance in the selection of optimal biosensor conditions. As the amount of donor DNA decreases, the fraction of donor fluorescence diminishes, reaching similar levels to the background cellular autofluorescence (green vs. gray wedges, Fig. 1c). As background fluorescence becomes more significant, we observe a decreased signal-to-noise ratio, resulting in the observed increased variability in the FLT signal.

We observed a similar trend in expression levels via flow cytometric analysis of αSN inter- and intra-protomeric cellular biosensors (Supplementary Fig. 4). For the inter-protomeric system, there is a strong acceptor signal that continues to accumulate with increasing titration. For the intra-protomeric biosensor, the 1:1 expression profile is clearly visible as the strong diagonal in the second quadrant. The results are further supported with western blot analysis of biosensor cell lysates from the D:A titration (Fig. 1d), which illustrates distinct bands for both GFP-αSN and αSN-RFP constructs (see Supplementary Fig. 5a for loading control for Fig. 1d).

In addition to full-length αSN fusion constructs, we observe both C-terminally and N-terminally truncated αSN species. These were confirmed by western blot analysis of total αSN (Syn1)^53^ vs. full-length αSN (LB509)^54^ along with green fluorescent protein (GFP) and red fluorescent protein (RFP) immunoreactivity (Supplementary Fig. 6). This effect is more observable in cells expressing the GFP-αSN-RFP and αSN-RFP fusion where the C-terminal truncation (resulting in Syn1-positive but not RFP-positive bands) is more prominent than N-terminal (Syn1-positive, LB509-positive, GFP-negative bands). The presence of αSN truncation products biased toward C-terminal truncation motivated the use of an N-terminal fused donor. In these FRET biosensors, any protein cleavage that results in free donor fragments would have a similar FLT signal as soluble GFP, reducing the observed FRET signal window. In contrast, due to the abundance of expression, free acceptor XFP fragments do not negatively impact FRET to the same extent, as only donor FLT is monitored.

The conclusion of these assays narrowed the selection of a D:A DNA ratio of 1:8, which optimized the FLT/FRET signal window and minimizes FLT signal variability.

### αSN biosensor characterization

**Fig. 2:**
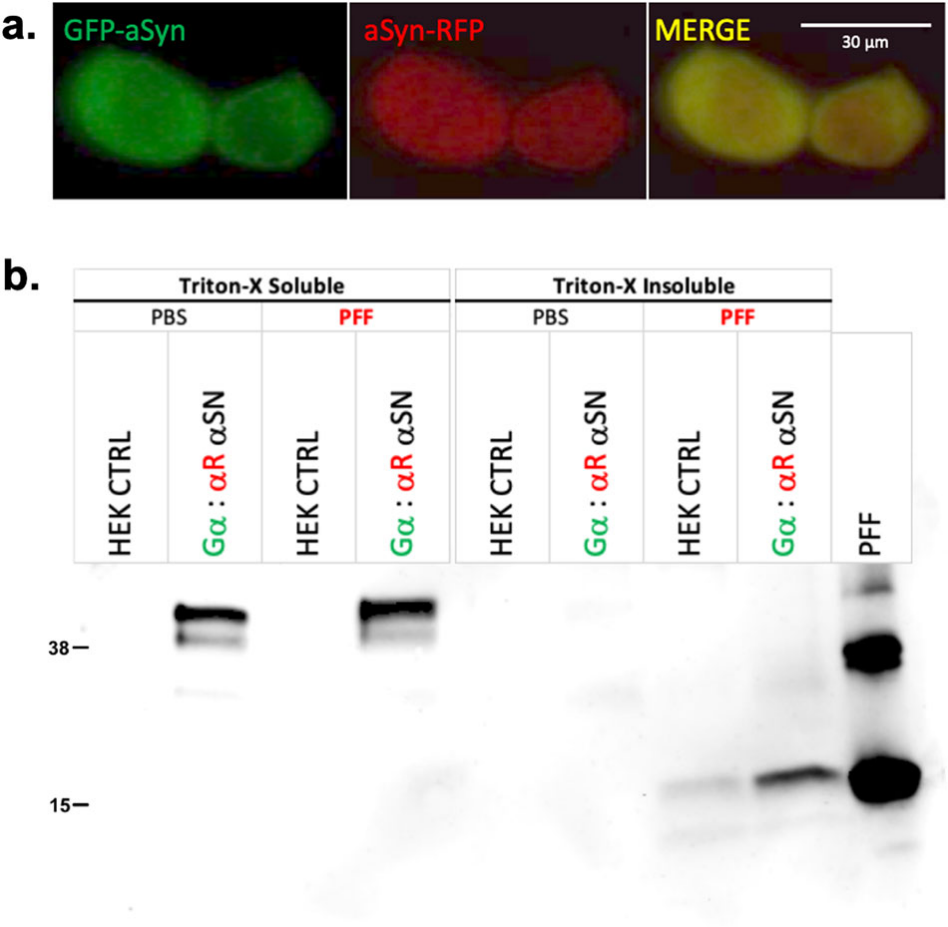
Characterization of αSN cellular FRET biosensors. **a** Fluorescence live cell imaging of our inter-protomeric cellular FRET biosensors co-expressing GFP-αSN/αSN-RFP constructs illustrate a diffuse expression profile (non-punctate). **b** To explore whether our biosensor FRET stems from soluble oligomers or fibrillary αSN, both control (HEK CTRL) and biosensor (Gα:αR αSN) transfected HEK cells were treated with PFFs (400 ng) or vehicle (PBS) for 24 h. Immunoblots of Triton-X soluble and insoluble with 2% SDS extraction were probed with Syn1 antibody and showed that all biosensor fusion constructs were extracted in the soluble fraction for both PBS and PFF conditions, indicating that soluble protomer/oligomeric αSN is present in our αSN biosensors. From the blots, insoluble αSN derived from PFF is detected in PFF-treated TX-100 insoluble fraction of cell lysate. See Supplementary Fig. 5 for loading control.

Our αSN FRET cellular biosensor screening platforms are engineered to target toxic, spontaneous αSN oligomers and conformations. It is therefore important to characterize the types of protein–protein assemblies present in our cellular biosensor. Fluorescence microscopy (Fig. 2a), western blot analysis (Fig. 2b and Supplementary Figs. 6–8), and cell-seeded ThT aggregation assays (Supplementary Fig. 9) provide insight into some of the direct protein–protein interactions present and were used to verify that our biosensors are monitoring early-stage αSN oligomers, and not fibrils. Figure 2a shows cells co-transfected with GFP-αSN/αSN-RFP, where the expression pattern is notably diffuse, devoid of puncta indicative of aggregated (fibrillar) αSN.

We then explored whether the FRET observed in our biosensors is derived from soluble oligomers or fibrillar αSN by treating control (CTRL) and biosensor (Gα:αR αSN) transfected HEK cells with PFF or vehicle (phosphate-buffered saline (PBS)) for 24 h. Since we are targeting pre-fibrillar oligomers, we focused on shorter transfection and treatment regimes. Previous studies have shown that fibrillar αSN aggregates accumulate in detergent-insoluble fractions^55–59^, especially when induced by PFF seeds. Immunoblot analyses of Triton-X soluble and insoluble (with 2% SDS extraction) fractions showed that all the biosensor fusion constructs were extracted in the soluble fraction for both PBS and PFF treatment, indicating that soluble protomer/oligomeric αSN is present in our αSN biosensors. The experimental conditions for our αSN biosensor HTS are more comparable to PBS treatment; as seen in (Fig. 2b), there is minimal insoluble/fibrillar αSN in the PBS-treated samples (loading control for Fig. 2b presented in Supplementary Fig. 5b). Only treatment with PFF resulted in insoluble αSN in the Triton-X insoluble fraction, where control cellular lysates showed only a modest increase in the amount of detergent-insoluble αSN(m) and Ga:aR αSN cells exhibited a threefold increase in the amount of Triton-X-insoluble αSN(m) over controls (Fig. 2b). This shows that PFF addition is required for αSN recruitment into the insoluble fractions. Our short PFF treatment regimen is within the 2–4-day recruitment lag phase, where we observed a lack of recruitment of αSN biosensor constructs into PFF-induced puncta, as seen in other studies^58,59^.

We next determined whether our cellular αSN biosensor are phosphorylated at Serine-129 (pS^129^). Accumulation of pS^129^ is a hallmark of PD pathology. Supplementary Fig. 7 presents immunoblot analysis of biosensor cell lysates probed for pS^129^ (left) or total αSN (right) with unlabeled αSN and αSN PFF used as control samples for positive pS^129^ staining. We observed strong pS^129^ reactivity with the unlabeled and GFP-αSN sample; however, both inter- and intra-protomeric biosensors have a greatly reduced pS^129^ signal. This suggests that our current biosensor platform is predominantly αSN assemblies without pS^129^.

The robust FRET signal in the inter-protomeric biosensor suggests that direct αSN–αSN interaction is present in the biosensor cells. Using immunoprecipitation (IP) (Supplementary Fig. 8), we isolated soluble GFP or GFP-αSN from HEK293 cell homogenates and probed for the pull-down of αSN or RFP to indicate direct αSN–αSN interactions or XFP-induced artifacts, respectively. Supplementary Fig. 8 shows that GFP-αSN is able to pull down endogenous unlabeled αSN while lysate from control cells and cells expressing soluble GFP or RFP do not. Although these IP experiments were not conclusive in resolving direct protein–protein interaction between GFP-αSN and αSN-RFP fusion constructs in the inter-protomeric biosensor, our results do show that non-specific interactions between soluble GFP and αSN are not observed either. Due to the early stage of αSN assemblies in these biosensors, it is plausible that the protein–protein interactions which are responsible for FRET are not sufficiently mature to survive the IP assay conditions.

Next we explored whether or not the inter-protomeric αSN biosensor constructs contain seeding-competent αSN assemblies. Supplementary Fig. 9 presents cell lysate-seeded ThT aggregation assay results for a series of HEK293 cell lysate conditions (untransfected, unlabeled αSN, and inter-protomeric biosensor) with and without recombinant αSN or PFF seed. Indeed, only samples that had both recombinant αSN(m) and PFF seed displayed positive ThT fluorescence. The biosensor-expressing lysate was indistinguishable from control HEK cell lysate results. This confirms that the αSN assemblies responsible for producing FRET are not seeding competent and therefore are pre-fibrillar in nature. Taken together, these results provide evidence that our FLT αSN biosensor experiments are conducted in conditions that do not promote fibrillization of αSN unless induced with αSN PFFs.

### αSN biosensor sensitivity

**Fig. 3:**
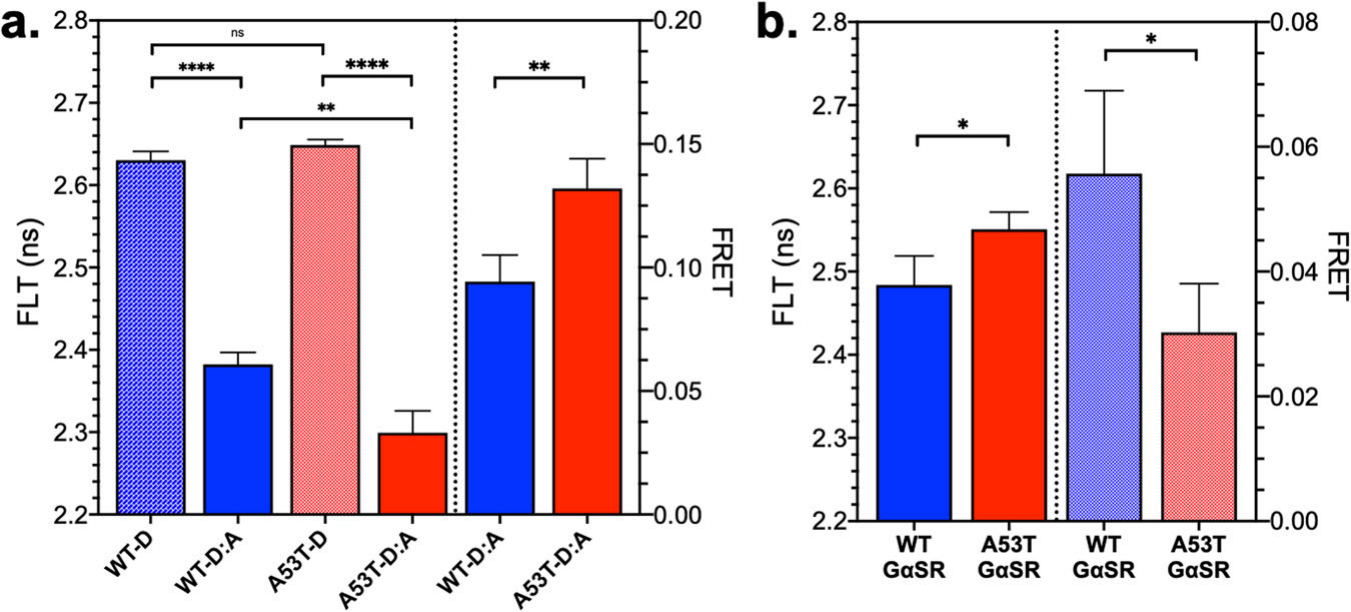
Characterization of αSN cellular FRET biosensors. **a** Comparison of WT and A53T αSN inter-protomeric FRET biosensors show decreased fluorescence lifetime and corresponding increased FRET for the aggregation-prone A53T construct relative to WT. **b** Comparison of WT and A53T αSN intra-protomeric FRET biosensors shows an increased fluorescence lifetime and decrease in FRET for the A53T relative to WT. Experiments were done with *n* = 5; *p < 0.05, **p < 0.01, ****p < 0.0001, ns indicates no significant difference as determined by Student’s *t* test.

As a first test of the sensitivity of this platform, we also developed a set of mutant A53T αSN biosensors. The A53T familial mutant is known to increase aggregation propensity and toxicity^60–64^. Comparison of WT and A53T inter- and intra-protomeric biosensors demonstrate significant FLT and FRET differences (Fig. 3a, b, respectively). As expected, the aggregation-prone A53T mutant resulted in increased FRET relative to WT for the inter-protomeric biosensor. This agrees with previous findings by Tosatto et al. that cell-free aggregation of A53T αSN oligomerizes more rapidly than WT and preferentially matures into a more compact, β-sheet-positive oligomer with increased inter-protomeric FRET^65^. Interestingly, in the intra-protomeric biosensor, the mutant induced a reduction in FRET, suggesting a more extended conformation for A53T, highlighting the sensitivity of these biosensors to changes in αSN conformation in these complex, heterogeneous assemblies.

We next looked into the relative D:A expression levels for both WT and A53T biosensor systems. Although the intrinsic nature of FLT is a major advantage for FLT-FRET biosensors, during their development it is essential to characterize biosensor expression for an aggregation-prone protein like αSN because more αSN can drive increased aggregation. Using SUPR experiments on both WT and A53T inter-protomeric biosensor, we observed a consistent and significant increase in D:A ratio for A53T relative to WT (Supplementary Fig. 10a–c). This complicates the interpretation of the increased FRET in A53T, as it may be due to differences in expression as well as increased aggregation propensity. However, comparison of SUPR fits from the intra-protomeric biosensors (Supplementary Fig. 10d–f) revealed that both WT and A53T have similar expression profiles. The intra-protomeric construct ensures a 1:1 D:A ratio, improving consistency across biosensor variants, with XFP folding and maturation being primarily responsible for fluorescence differences. Even with the SUPR discrepancies in the inter-protomeric biosensor comparison, these experiments do show that our technology is sensitive to small changes in αSN sequence and confirms the capacity to monitor structural changes in an intrinsically disordered protein system.

**Fig. 4:**
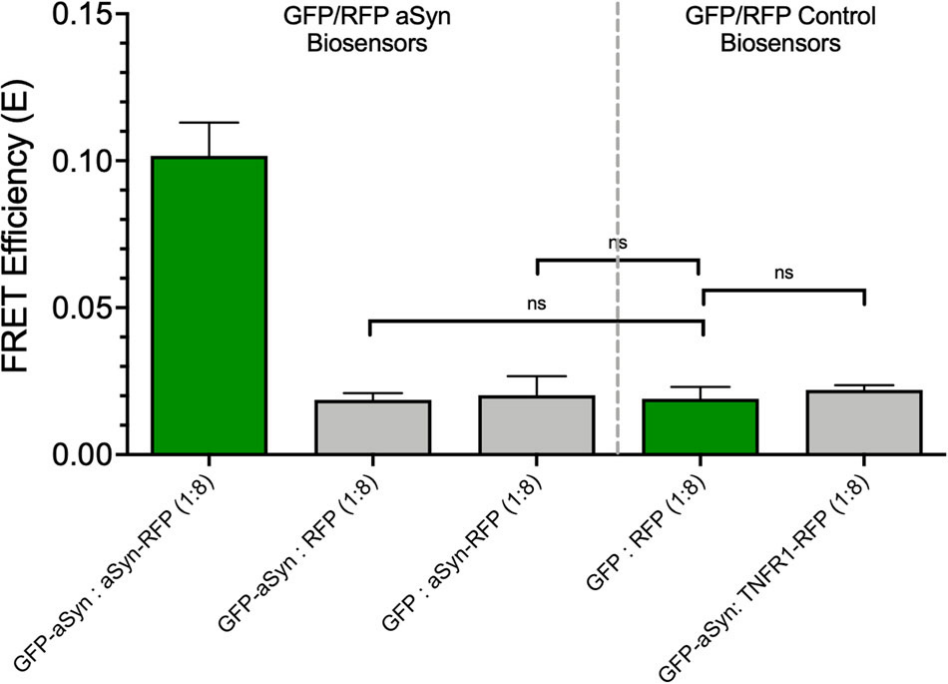
XFP fusion does not contribute significantly to background FRET signal. One of the major concerns with any tagged or fusion protein assay are the potential of non-specific protein–protein interactions being driven via the XFP or fluorescent tag. Here we compared our inter-protomeric αSN cellular FRET biosensor with soluble mEGFP/TagRFP at the same D:A ratio as well as hetero-biosensor combinations to explore potential non-αSN-driven FRET. GFP-αSN to soluble RFP, soluble GFP to αSN-RFP, and GFP-αSN to TNFR1-RFP systems demonstrate little to no difference from soluble GFP/RFP systems. We conclude that the significant FRET in the inter-protomeric biosensor relative to soluble GFP/RFP is therefor due to direct αSN–αSN interactions. ns indicates no significant difference as determined by Student’s *t* test.

The addition of XFP fusion proteins to any cellular system can induce artifacts and changes in the target proteins’ native function. To further evaluate our biosensors’ sensitivity, we compared the αSN inter- and intra-protomeric biosensors to a series of control biosensors to investigate the potential for non-specific interactions being driven by the XFP fusion. Figure 4 presents a comparison of the αSN inter-protomeric (D:A 1:8) to a series of control biosensors, including soluble GFP/RFP (D:A 1:8), GFP-αSN to soluble RFP, GFP-αSN to TNFR1-RFP, and GFP to αSN-RFP. We observe a consistent FRET efficiency of ~0.02 for control GFP/RFP (D:A 1:8) biosensor system with no significant differences for other non-specific FRET biosensors. A comparison of intra-protomeric αSN and control biosensor is presented in Supplementary Fig. 11. The significantly larger FRET from our inter-protomeric GFP-αSN:αSN-RFP system provides a large αSN-dependent FRET signal window for use in our HTS campaigns.

### Pilot HTS with the LOPAC library for the oligomer and monomer conformation αSN FRET biosensors

Prior to each pilot HTS, FLT and emission spectrum were measured to verify signal level, basal FRET, coefficient of variance (CV), and similarity index. For each HTS, cells were dispensed into compound plates—1536-well (10 µL/well)—and incubated with the compounds (10 µM) or dimethyl sulfoxide (DMSO; 1%) for 90 min. FLT measurements were acquired with the FLT-PR. After deconvolving the instrument response function, a single exponential fit was used to determine the FLT for both the αSN cellular FRET biosensor (*τ*_DA_) and the donor-only control biosensor (*τ*_D_). FRET efficiency was determined by Eq. 1. CV was determined from control plates (*N* = 384 wells).

**Fig. 5:**
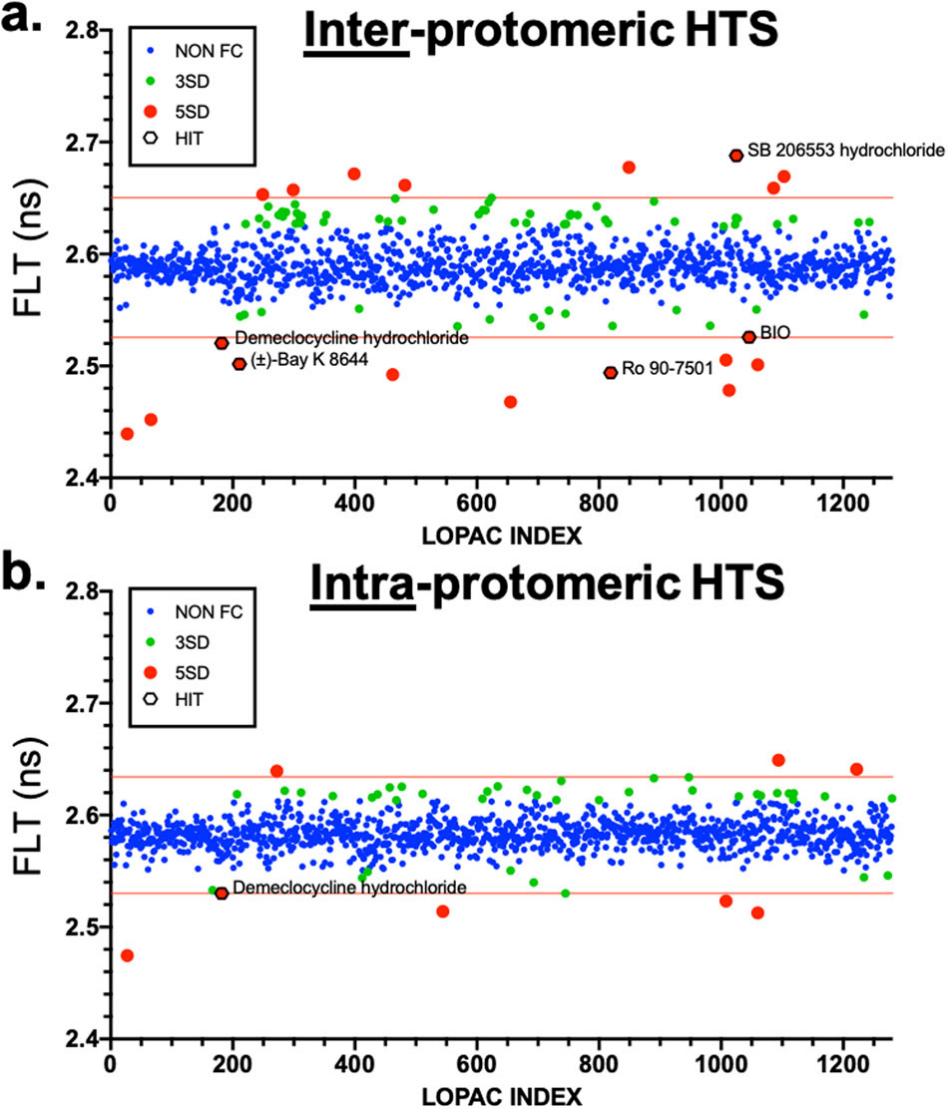
Pilot HTS on cellular αSN FRET biosensors. **a**, **b** Screens of the 1280-compound LOPAC library were performed in a single 1536-well plate for both the inter-protomer (**a**) and intra-protomeric (**b**) biosensor. Pilot HTS provided 19 and 8 hits, respectively, that changed FLT by >5 SD (red). For some systems, a 3-SD (red+green) threshold is sufficient if secondary assays can process the increased throughput.

A major challenge to any fluorescence-based HTS platform stems from optically interfering (e.g., fluorescent) compounds in the library. Fluorescent compounds (FC) were flagged using a spectral similarity index filter, which evaluates the ratio of intensity from two bandwidths that span the donor emission spectrum and are excluded as potential false positives^45,46,49,51,66^. FLT values for all compounds that passed the FC filter are presented in Fig. 5 for both the inter-protomeric (Fig. 5a) and intra-protomeric (Fig. 5b) biosensor HTS. Each compound plate included 256 DMSO control wells as a control for the αSN FRET biosensor signal quality over time. Both biosensor HTS datasets exhibited a tight Gaussian distribution of compounds (Supplementary Fig. 12). Compounds that changed FLT by >5 standard deviations (SD) were considered hits (Fig. 5, red data points). The 5-SD threshold is an arbitrary definition that was made to reduce the number of compounds that are investigated in subsequent secondary assays. In total, 21 hits were explored, 19 hits from the inter-protomeric screen and 8 hits from the intra-protomeric biosensor at the 5-SD threshold (with 6 overlapping compounds identified in both screens, see Supplementary Table 1).

The screen identified multiple compounds within the LOPAC library that were previously known to attenuate αSN-induced toxicity: AGK2 (a SIRT2 antagonist) and Bio (an autophagy inducer)^67,68^, as well as those shown via BiFC (e.g., DEM, PD173952, PD169316)^42^. DEM was not pursued as a functional inhibitor in the BiFC study due to the potential interference of the tetracycline derivative compound with their Tet-Off expression system. Additional hits in our screen span a range of known pharmacological functions that directly affect cellular pathways known to be disrupted by αSN overexpression (e.g., modulating oxidative stress, reducing inflammation, inhibiting nitric oxide synthase, and upregulating autophagy). The identification of known effectors of αSN pathology as well as compounds relevant to αSN-induced cellular dysfunction reinforce the efficacy, sensitivity, and selectivity of our αSN cellular FRET biosensor.

FLT changes in both inter- and intra-protomeric biosensors may indicate of a wide range of conformational changes (Supplementary Fig. 1). An increase in the basal inter-protomeric FLT (decreased FRET) could result from the dissociation of spontaneous formed oligomers or through structural rearrangement of oligomers that shift the ensemble of donor-to-acceptor XFP distances further apart. Similarly, a reduction in FLT (increased FRET) could be due to increased oligomerization (e.g., a shift in the equilibrium between monomer and soluble, non-toxic tetramer^19,24^) or structural rearrangement toward a more compact oligomer. The intra-protomeric double-fusion biosensor provides complementary insight into αSN, as FLT changes can arise from changes in oligomerization or in monomer conformation, where reduced FLT corresponds to a more compact ensemble of conformations.

### FLT dose response of hits with WT and A53T αSN FRET biosensors

**Fig. 6:**
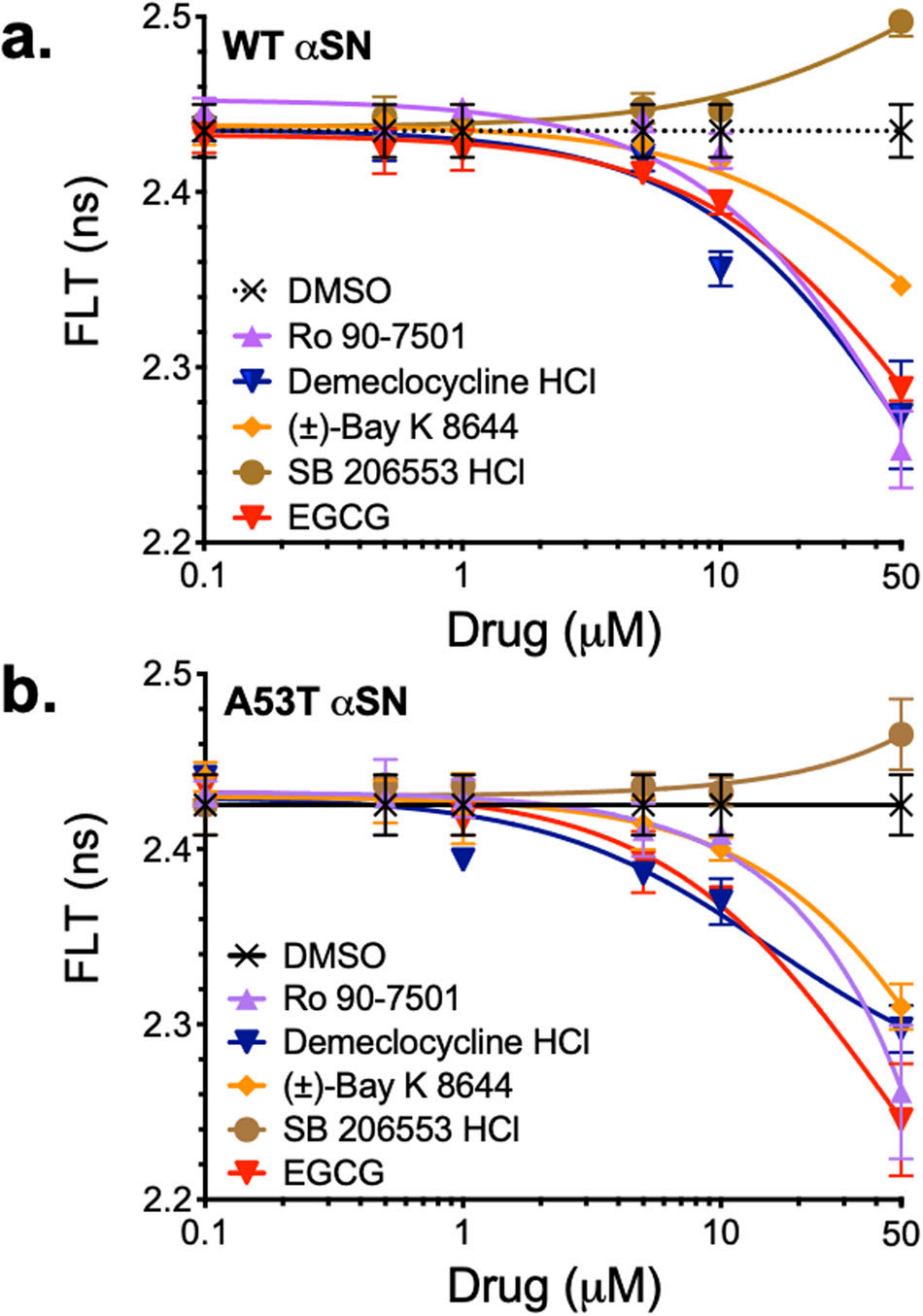
FRET dose response of pilot HTS hits. A subset of hits were evaluated using both WT (**a**) and A53T (**b**) oligomer biosensor to verify both increased FLT (disrupting αSN–αSN interactions) and decreased FLT (tighter aggregation or remodeled oligomers). Three hits that reduced FLT (RO, BAY, DEM) demonstrated similar response as control EGCG, whereas hit SB206553 demonstrated mild recovery of FLT, suggesting dissociation.

Hits from the pilot HTS of the LOPAC library were selected and dispensed into 384-well plates to verify reproducible and specific FLT trends using the WT and A53T oligomer (inter-protomeric) FRET biosensors. Figure 6 presents a subset of these dose-response curves for hit compounds RO, BAY, SB206553, and DEM, along with known control compound EGCG. For both WT and A53T biosensors, these compounds displayed similar dose-response signal. Concentrations above 50 µM showed significant cytotoxicity for some compounds, prohibiting an accurate FLT IC_50_ determination. Of the hits tested, only SB206553 showed an increasing FLT trend, suggesting dissociation or loosening of monomers within oligomers (decreased FRET). All other hits (including control compound EGCG) displayed a reduced donor FLT (increased FRET), indicative of increased oligomer formation and/or remodeling of oligomers into a more compact conformation. Assay quality was determined by *Z*′ (Eq. 2) where a *Z*′ > 0.5 indicates good assay quality. Using EGCG as control compound and the hit DEM, we calculated *Z*′ value of 0.76 and 0.66, respectively, indicating excellent assay quality.

**Fig. 7:**
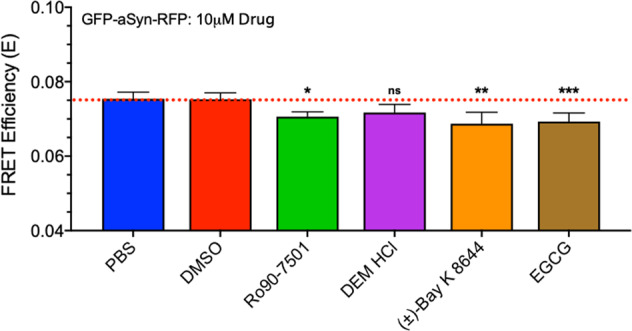
FRET response to pilot HTS hits on intra-protomeric αSN biosensor. Hits that demonstrated robust, reproducible FRET response in the inter-protomeric biosensor CRC were then evaluated with the intra-protomeric αSN biosensor. With both donor and acceptor XFP fused to one construct, this biosensor is sensitive to both oligomer formation as well as conformation. Three hits reduced the intra-protomeric FRET (RO, BAY, DEM) significantly, as did known αSN modulator EGCG. Experiments were performed with *n* = 3; *p < 0.05, **p < 0.01, ***p < 0.001, ns indicates no significant difference as determined by Student’s *t* test.

Hits were also evaluated with the intra-protomeric αSN and control, non-specific biosensors. Figure 7 illustrates that our hit compounds induced a significant change in FRET at 10 µM in the intra-protomeric αSN biosensor. The compounds RO, BAY, and DEM as well as the control EGCG each reduced FRET, indicating either reduction in oligomerization or expanded oligomer conformation. Tests of these compounds in two control biosensor systems (GFP/RFP at 1:8 and GFP-linker32-RFP; see Supplementary Fig. 13a, b, respectively) show no significant FRET change. These control biosensors provide support that the hit compounds have an αSN-dependent effect and do not modulate the XFPs directly. Interpreting these results in light of the inter-protomeric concentration–response curve (CRC) suggests that these hit compounds are changing the oligomer structure and not dissociating oligomer assemblies.

### Secondary assay: total cellular viability

We note the significant potential for artifacts due to the labeling of αSN with large synthetic fluorophores (fusion constructs add ~25 kDa of XFP onto a 15 kDa protein). Thus, all secondary cellular assays presented below (cytotoxicity, expression, pathology) are done with unlabeled αSN. It is also important to note that we cannot determine, a priori, whether an increase or decrease in FLT correlates to reduced toxicity.

**Fig. 8:**
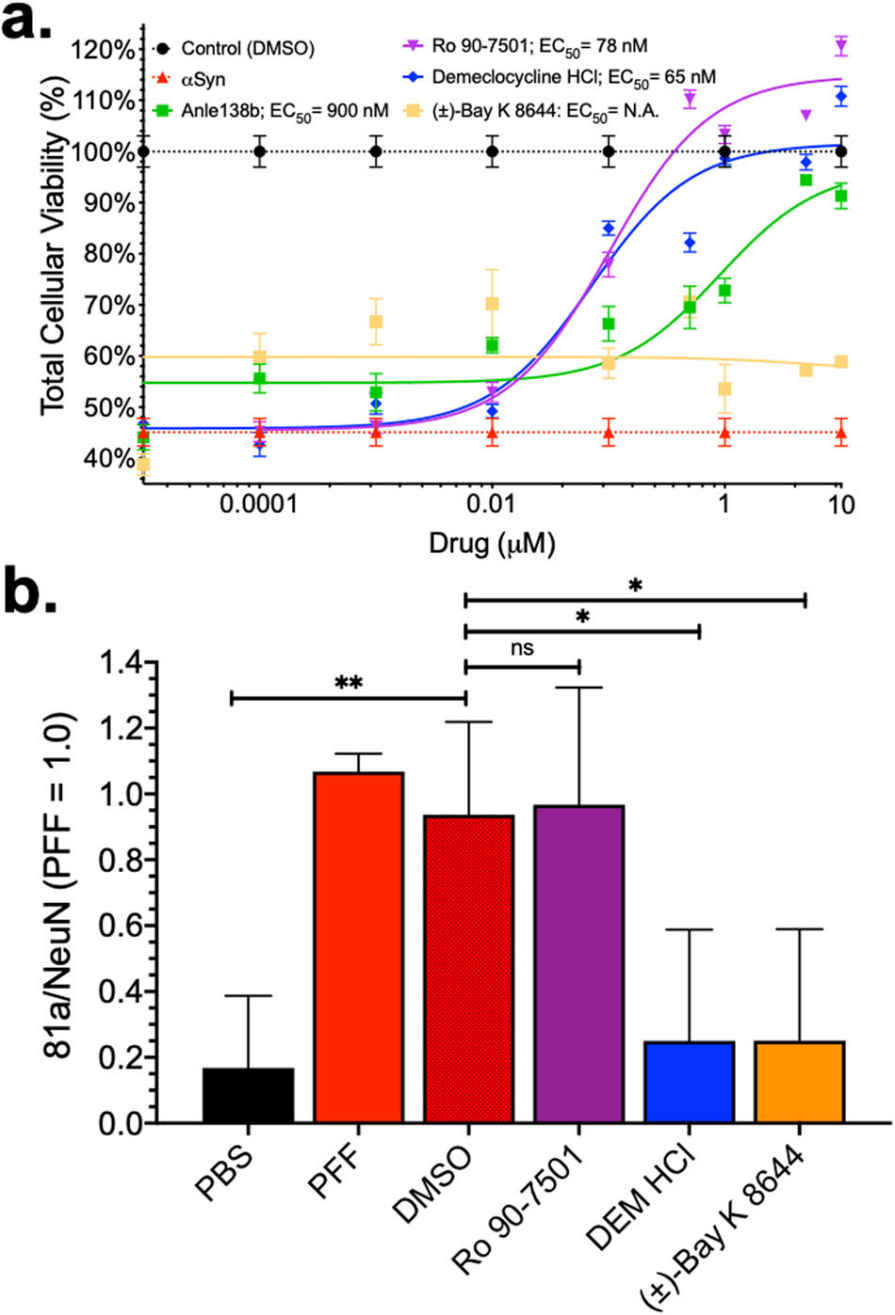
Cytotoxicity in SH-SY5Y cells and primary neuron pathology model demonstrate hit compound rescue of αSN induce pathology. **a** Overexpression of αSN in SH-SY5Y cells results in reduced cellular viability (~44%, red dashed line) as determined by CytoTox-Glo cytotoxicity assay. Treatment with control compound Anle138b as well as hit compounds RO and DEM demonstrate a dose-dependent rescue of toxicity with an EC_50_ of 900, 78, and 65 nM for Anle138b, RO, and DEM, respectively. Biosensor hit BAY did not rescue the SH-SY5Y cell toxicity. **b** Using an αSN PFF-induced pathology model in primary mouse neurons, we evaluated the capacity of our hit compounds to rescue αSN PFF seed-associated pathology (phospho-S^129^ staining). PFFs were treated at ~415 ng. Two of our hit compounds reduced the αSN pathology (BAY and DEM). Representative micrographs of each system are presented in Supplementary Fig. 14 with quantification of cytotoxicity via NeuN counting in Supplementary Fig. 15. *N* = 3 independent experiment; *p < 0.05, **p < 0.01, ns indicates not significant.

Hit compounds were evaluated for rescue of αSN-induced cytotoxicity in using an SH-SY5Y neuroblastoma cell model of alpha-synucleinopathy^18,69–72^. Overexpression of WT αSN showed significant reduction in cell viability (46% total viability, Fig. 8a and Supplementary Fig. 14). There is clear dose-dependent rescue of toxicity for hits RO and DEM, with reduced effect for BAY. Although hit SB206553 demonstrated reproducible and dose-dependent increased FLT (Fig. 7), there was no observed effect on cytotoxicity, highlighting that a change in FLT does not necessarily correlate with changes in toxicity. A seven-point CRC for RO, BAY, DEM, and Anle138b (positive control compound^41,73^) was performed to determine IC_50_ for each compound. Both RO and DEM demonstrated potent inhibition of αSN-induced toxicity with an IC_50_ of 78 and 65 nM, respectively (Fig. 8a).

We note that these IC_50_ values, which are an order of magnitude more potent than previously published small-molecule inhibitors, are significantly lower than the lifetime dose responses. There are several possible explanations for this: they are performed in different cell lines (HEK293 vs. SH-SY5Y), under different treatment conditions (FRET is measured after a 48-h transfection and 20–90 min compound exposure, whereas cytotoxicity assays are conducted with a 72-h transfection and 48-h exposure to the compound.), with different constructs (+/− FRET labels) and with different phenomena being measured. (FRET measures changes in distance with an *R*^−6^ dependence, with picosecond FLT resolution vs. cytotoxicity, which measures bulk lactate dehydrogenase enzyme released in media relative to total cell via luminescence accumulated over 0.5 s). It is also possible that the effect in SH-SY5Y cells is more pronounced due to interactions of the compounds with other protein machinery involved in αSN-induced cell death. Indeed, DEM is a known calpain inhibitor^74^ and can play an active role in ameliorating calpain-dependent NLRP3 inflammasome activation, a known effector of αSN-induced pathology^75–80^. The interplay between potential MOA, whether direct binding of compounds to αSN or indirect MOA (e.g., via inhibition of calpain), are discussed at length below.

We investigated one potential MOA for compounds RO and DEM in our SH-SY5Y cytotoxicity model. These compounds might rescue αSN-induced cytotoxicity by modulating αSN expression^81,82^. SH-SY5Y cells transfected with empty-vector or unlabeled WT αSN were treated with DMSO, RO, or DEM at 1 μM concentration. Supplementary Fig. 15 shows that the drug treatment interestingly increases total αSN expression levels. This effect may be due to prolonged survival of cells expressing higher levels of αSN, which would be lost in DMSO-treated samples or as a result of shifting monomer:oligomer ratios^82^. Studies have shown that there is a fine balance in αSN expression, where too much can lead to aggregation, but αSN expression can also be neuroprotective^81,83–86^.

### Secondary assay: primary neuron model of αSN PFF pathology

Further analysis of hits RO, BAY, and DEM in an αSN PFF model also exhibited some protection against later-stage αSN pathology. As described previously, numerous in vivo and in vitro alpha-synucleinopathy mouse models have been developed using treatment with sonicated αSN PFF to model pathology. PFF treatment of primary mouse hippocampal neurons results in neurotoxicity and increased phosphoserine-129 (pS^129^) immunoreactivity, a marker for alpha-synucleinopathy pathology^87,88^. These effects can be produced using either human-PFF or murine-PFF with the murine-PFF providing a stronger response. Primary neurons (7 days in vitro (DIV)) were treated for 12 days with murine-PFF or PBS and 1 µM hit compound or DMSO (compound vehicle). Neurons were fixed and stained for NeuN (neuronal nuclei), Map2 (neurite and neuronal morphology), and 81a (pS^129^) (see Supplementary Fig. 16). PFF treatment results in a significant loss of neurons (NeuN count, Supplementary Fig. 17) and a significant increase in pathology staining (e.g., normalized 81a/NeuN) that is unchanged with DMSO treatment (Fig. 8b). Treatment with 1 µM of either BAY or DEM reduces the pS^129^ phenotype; however, treatment with RO had little to no effect. Reduction of pS^129^ signal in our co-treatment model (e.g., where both PFF and compound are administered simultaneously) could suggest either a rescue of PFF-induced pathology or inhibition of the pS^129^ signal through reduction of endogenous αSN phosphorylation. Future studies focused on hit-to-lead optimization will focus on these nuances to resolve hit compound’s MOA.

### Secondary assay: differentiated SH-SY5Y cell model of αSN PFF pathology

We next explored an orthogonal PFF pathology model that monitors pS^129^ signal in differentiated SH-SY5Y cells treated with sonicated human αSN PFF and show significant effects for each compound (Supplementary Fig. 18). We observe a distribution of pS^129^ immunoreactive bands that we delineate as monomeric, oligomeric, and high-molecular-weight (HMW) αSN species as well as a series of non-specific bands that are present across all samples (Supplementary Fig. 18a, b). Quantifying total pS^129^ (excluding non-specific bands) shows a significant decrease in total pS^129^ signal for RO, a significant increase in total pS^129^ for BAY, and a non-significant change for DEM (Supplementary Fig. 18c). Obvious differences between these data and those from the primary mouse neurons are noted here and discussed below. Parsing the data more closely, Supplementary Fig. 18d shows that all three compounds strongly alter the relative ratios of the various pS^129^ assemblies. In particular, all three hits drove a reduction in monomeric pS^129^ and an increase in HMW pS^129^. Interestingly, two of the three hits (RO, DEM) and a control compound (Baicalin) also significantly reduced the low-molecular-weight (LMW) oligomer fraction.

### Secondary assay: interactions with αSN monomers

Results from our intra-protomeric αSN biosensor FRET response suggested that hit compounds RO, Bay, and DEM may be modulating αSN oligomers by changing their conformation. The intra-protomeric biosensor is unable to distinguish between changes in monomer or oligomer conformation. To investigate the effects of our hit compounds on monomer conformation, we used smFRET and PrOF NMR. These biophysical techniques are performed under conditions where αSN is predominantly monomeric.

SmFRET experiments were performed using two different labeled αSN constructs (residues 9–72 and residues 54–130) that report on intra-monomeric FRET (e.g., monomer conformation) of the N-terminal and C-terminal domains of αSN. Neither Bay nor DEM showed a significant change in smFRET histograms (Supplementary Fig. 19 and Supplementary Table 2) indicating that these compounds do not modulate monomeric conformation. We were not able to evaluate RO with smFRET, due to interactions of RO with the AlexaFluor-594 label.

PrOF NMR provides a perturbation-free method to monitor αSN conformation and structure through the incorporation of F19-labeled 3-fluoro-Tyrosine into recombinant protein^89^. The resting PrOF spectra for F19-labeled αSN shows three resonances that have been previously assigned to the four tyrosine residues (Supplementary Fig. 20a)^89^. Titration of hit compounds RO and DEM with fluorinated αSN shows no significant chemical shifts or resonance broadening (Supplementary Fig. 20b, c) except for high concentrations of RO (in excess of 5:1 compound-to-protein ratio). These results support a lack of binding to αSN(m). Results from both smFRET and PrOF NMR suggest that the potential MOA for these hit compounds is likely not via direct binding or interaction with αSN(m).

### Secondary assay: modulation of αSN oligomers

**Fig. 9:**
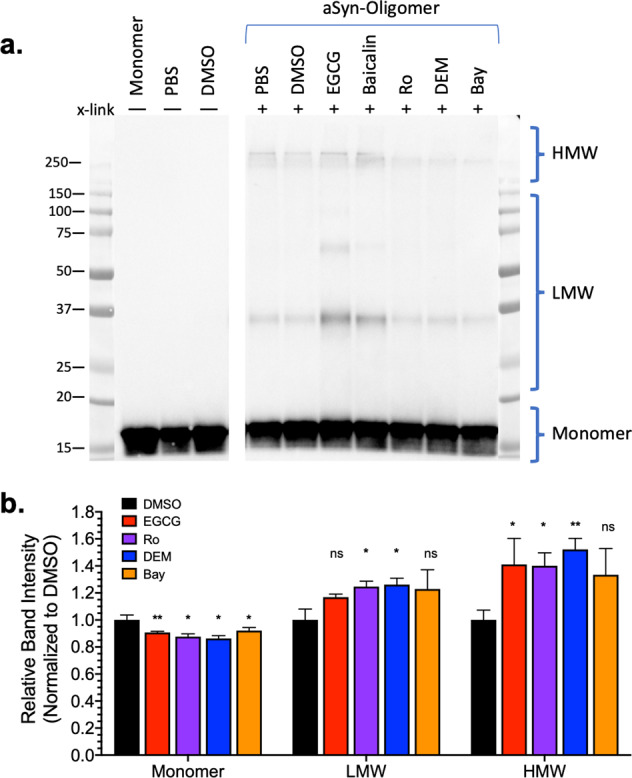
Recombinant αSN oligomerization assay. **a** Cross-linked aliquots from recombinant αSN oligomerization assay were ran via SDS-PAGE, transferred, and probed for total αSN with antibody Syn1. Samples before and after cross-linking were evaluated and densitometry was performed by classifying αSN assemblies as monomer, low-molecular-weight (LMW) oligomers, or high-molecular-weight (HMW) oligomers. **b** Densitometry analysis on cross-linked αSN oligomers (see Supplementary Fig. 18) illustrate a significant change in monomer (decrease) and both oligomers (increase) with compound treatment. All compounds were at sub-stoichiometric ratio (1:5, compound: monomer). *N* = 3 independent experiments; *p < 0.05, **p < 0.01, ns indicates no significant difference as determined by Student’s *t* test.

Next we explored our hit compounds effect on recombinant αSN oligomers via a modified αSN oligomer assay adapted from Otzen et al.^90^. Purified recombinant, αSN(m) (350 μM, 5 mg/mL) was incubated with each compound (70 μM; a 1:5 compound:protein ratio) for 12 h under continuous shaking (900 RPM, 37 °C). After oligomerization, an aliquot of each sample was cross-linked in 1.6% paraformaldehyde (PFA) and characterized via SDS–polyacrylamide gel electrophoresis (PAGE) western blot analysis^91^. Figure 9a shows the resulting products from our oligomerization assay for non-cross-linked and cross-linked samples. Using the cross-linking, we are able to investigate SDS soluble assemblies that would be otherwise dissociated. There are clear changes in the distribution of bands with different compound treatments. Experimental replicates were processed on the same blot (Supplementary Fig. 21), with densitometry quantification based on monomer, LMW oligomers, and HMW oligomers (Fig. 9b). Each species was normalized to the DMSO-treated sample. There was a significant decrease in monomer and subsequent increase in LMW and HMW oligomers for almost all hit compound conditions.

To explore the presence of β-sheet content in these oligomers, an aliquot was removed prior to cross-linking, diluted into 100 μM ThT working solution, and ThT fluorescence was monitored to detect the presence of β-sheet. Supplementary Fig. 22 presents each sample ThT content normalized to the PFF signal. We observed an increased ThT content in the αSN oligomers (αS-O) that was not affected by DMSO (vehicle) treatment. Both control compounds, EGCG and Baicalin, are known modulators of αSN aggregation and demonstrate a complete loss of ThT signal. The three hit compounds tested had either no effect (RO) or increased the ThT signal (DEM and Bay) over DMSO, albeit not to the same extent as PFFs.

These effects on αSN oligomers suggest that our hit compounds interact with αSN oligomers, increasing the overall oligomer fraction and modulate the corresponding αSN oligomer structure, supporting the finding from our inter- and intra-protomeric FRET biosensors.

### Secondary assay: inhibition of αSN fibrillization (non-seeded and seeded aggregation)

**Fig. 10:**
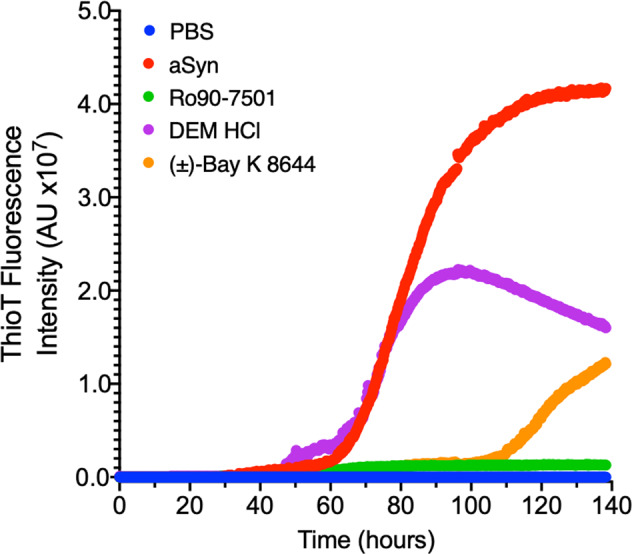
Monomer induced aggregation of αSN (non-seeded Thioflavin-T assay). Recombinant αSN was incubated at 140 μM monomer (70 μM compound) under continuous shaking (900 RPM) at 37 °C. All three hit compounds modulated αSN fibrillization with RO having a more significant effect over Bay and DEM, respectively.

ThT aggregation assays monitor the kinetics and extent of fibril formation due to spontaneous and seeded aggregation^26,92–95^. The cascade of spontaneous αSN fibrillization includes formation and maturation of oligomeric αSN assemblies^96,97^. Figure 10 presents spontaneous ThT aggregation for αSN(m) (140 µM) with or without hit compounds RO, DEM, and Bay (70 µM; a sub-stoichiometric ratio, 1:2, compound:protein). Here we observe significant ThT signal reduction for RO with lesser degrees of attenuation by Bay and DEM, respectively.

**Fig. 11:**
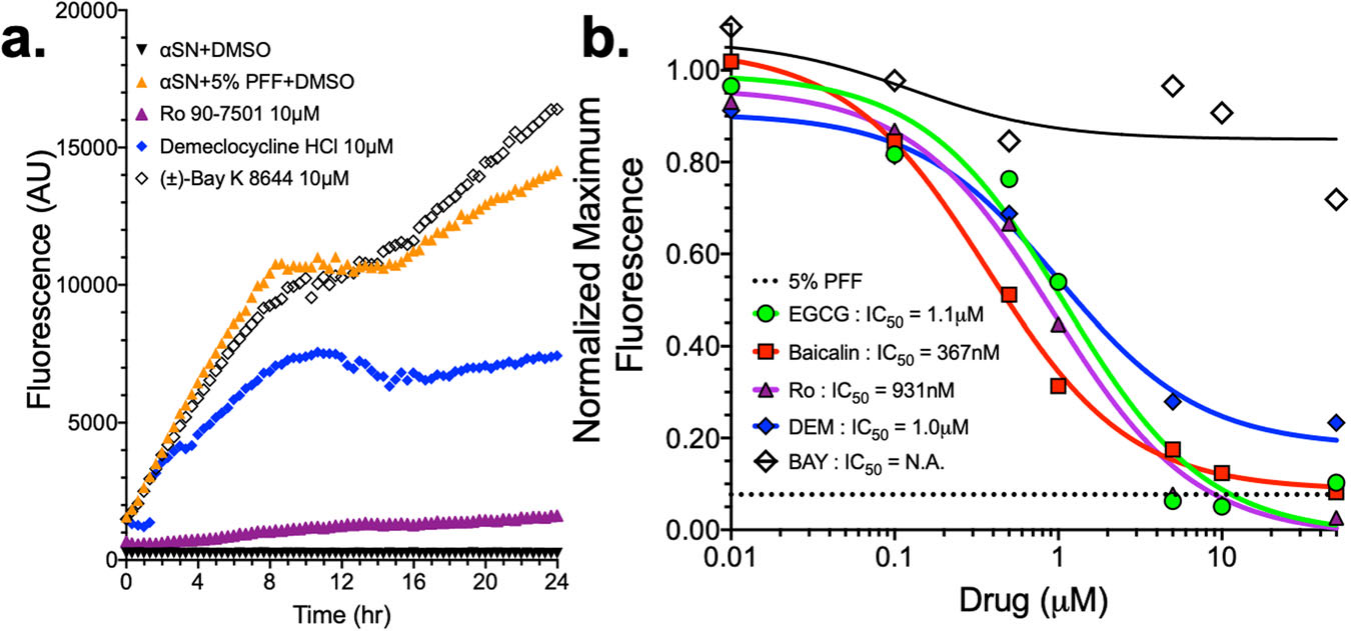
Seeded Thioflavin-T aggregation assay shows direct protein interaction for subset of hit compounds. Seeded ThT aggregation assay was performed using 15 μM monomeric αSN doped with 5% (0.75 μM sonicated αSN pre-formed fibril (PFF) seeds) in a 384-well plate under slow agitation (300 RPM) at 37 °C. **a** Control samples for the aggregation include PBS (black), αSN monomer (brown), 5% αSN PFF (red), and the 15 μM monomer+5% PFF mixture (orange). Dose-response curves were made with a titration of compounds. Some hit compounds had little to no effect on aggregation, even at 50 μM (e.g., SB206553). In contrast, RO and DEM both show potent effects relative to Baicalin and EGCG controls. BAY does have some response, but it lags behind other hit compounds. **b** Normalizing the initial peak in ThT data allows us to estimate an IC_50_ for each hit. Control compounds EGCG and Baicalin show 1.1 μM and 387 nM potency, whereas RO and DEM have IC50 of 931 nM and 1.0 μM. Although there is a small trend with BAY, the data cannot be fit to resolve an IC_50_.

Figure 11 and Supplementary Fig. 23 present results from a dose-response-seeded aggregation assay. Vehicle, control, and hit compounds were incubated with 15 µM αSN(m), doped with 5% freshly sonicated αSN PFF in PBS under mild agitation (Supplementary Fig. 23a). Seeded monomer samples underwent rapid elongation, whereas monomer-only samples did not spontaneous aggregate during the span of the experiment (Supplementary Fig. 23b). Titration of control and hit compounds were run in triplicate; representative traces of hit compounds are illustrated in Fig. 11a with a full summary of each titration detailed in Supplementary Fig. 23c–h. IC_50_ was determined for each compound by determining the max ThT intensity for each concentration (Fig. 11b). Both RO and DEM demonstrated similar IC_50_ as control EGCG (931 nM, 1.0 µM, compared to 1.1 µM for EGCG).

## Discussion

**Fig. 12:**
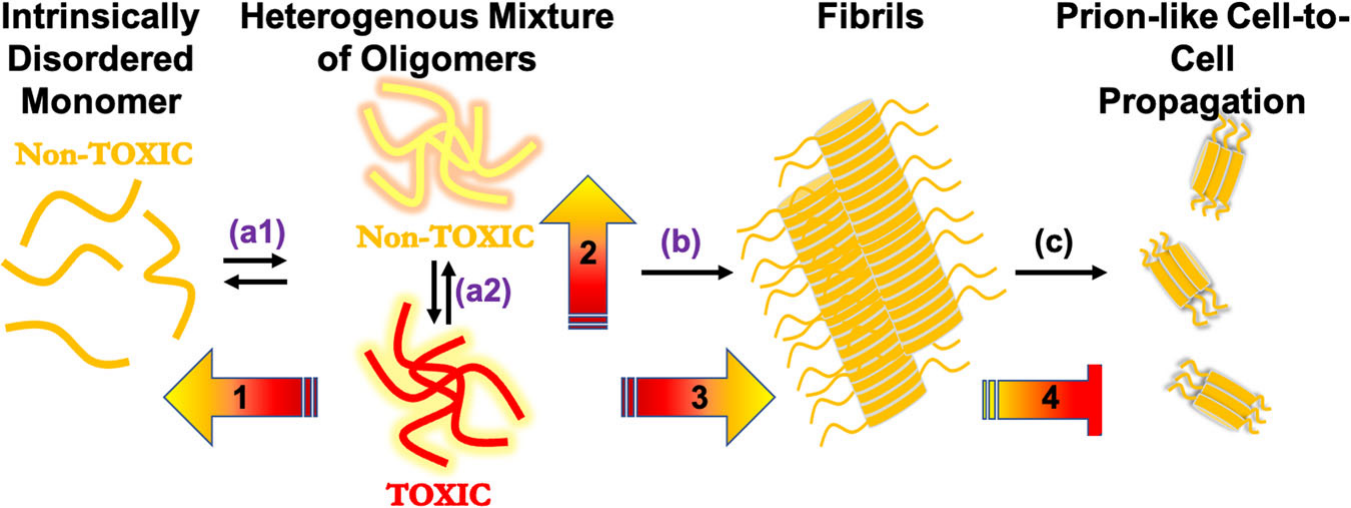
Monitoring spontaneous αSN oligomers and conformation using live cell fluorescence lifetime readouts. The intrinsically disordered αSN monomer is capable of misfolding into oligomers and fibrils, producing toxic assemblies that have been implicated in the pathology of disease. Oligomers comprise a heterogeneous mixture of metastable assemblies that have proven difficult to monitor with high precision and accuracy, so most current strategies target the irreversible formation of large assemblies (fibrils, arrow **b**). However, disruption of fibrils can induce toxicity due to elevated levels of toxic oligomers. Our cellular FLT-FRET biosensors monitor reversible spontaneous oligomerization (**a1**) in addition to structural remodeling within the oligomer assembly (e.g., conversion between toxic and non-toxic oligomers, **a2**), while also monitoring downstream processes such as fibrillization (**b**) and seeded aggregation (**c**) with high sensitivity. This study demonstrates the capacity of our drug discovery pipeline to identify compounds that modulate the toxicity of these αSN assemblies (gradient arrows 1–4), thereby shifting the equilibrium of these heterogeneous assemblies toward non-toxic species.

The folding/misfolding pathways that connect αSN monomers, oligomers, and fibrils (Fig. 12) are highly stochastic, and the degree of heterogeneity of molecular states may never be fully grasped. Even fibril structures are turning out to be variable, with distinct inter-monomeric motifs emerging under differing conditions and pathologies^98–102^. But the heterogeneity is of a different order, and is most confounding, for oligomers: do they have any discernable, stable structural motifs? Do these motifs contribute to the seeding competency of αSN assemblies? And, if so, are any of those motifs structured enough to constitute robust and reproducible small-molecule-binding sites? These uncertainties do not mean that small molecules targeting oligomers cannot act as high-potency inhibitors, as we have shown here that they can. Instead, the heterogeneous and metastable nature of αSN oligomers may force a re-thinking of conventional approaches to drug discovery. For example, rather than optimizing lead compounds to selectively and potently block an active site, we may need a cocktail of small molecules that act in a statistical way, collectively shifting the ensemble of accessible structural states of αSN to bias the folding pathway, shifting oligomers from toxic to non-toxic assemblies (Fig. 12).

A fundamental question arises from this framing: what is the ideal MOA for a small molecule, or a set of small molecules, that target αSN oligomers? One possibility is that the most efficient small molecules should directly bind αSN, in any of its myriad states, and perturb local structural elements—e.g., disrupts motifs or inhibit seeding—to prevent or reduce the population of toxic oligomers or spread of αSN pathology. This perspective is supported by a recent HTS campaign targeting SDS-induced αSN aggregation, where the most potent compounds predominantly targeted and bound to αSN(m), inhibiting subsequent aggregation^37^. We started from this traditional vantage point, assuming that our best hits would increase FLT (decreased FRET) through interacting with αSN(m). We based this on our experience with a similar screening platform developed to target tau oligomers, where our most functionally efficient hit decreased FRET^47^. To our surprise, the compounds with the most potent functional profiles (DEM and RO) increased FRET in our inter-protomeric biosensor, while the one hit that decreased FRET (SB206553) had no effect on cytotoxicity. It is possible that no small molecules in the LOPAC library, or any single compound for that matter, can fully dissociate or prevent oligomers. More likely, these small molecules either increase the extent of oligomerization or alter the organization of the protomers within the oligomers in such a way that (1) brings the fluorophores in closer proximity (possibly reflecting increased compaction) and (2) concomitantly buries or exposes motifs within the oligomer that both slow fibrillization (Figs. 10 and 11 and Supplementary Fig. 23) and reduce toxicity (Fig. 8 and Supplementary Figs. 14 and 17) in as yet unknown ways.

Determining whether these motifs exist, and what they are, may yet be possible. High-resolution structural measurements in cells are difficult, but progress is being made. For example, recent NMR and electron paramagnetic resonance experiments in cells have shown that αSN maintains disorder in the cellular milieu^103^. And deep mutational sequencing techniques may be useful in mapping a binding site^104^. The lifetime FRET measurements made here capture lower-resolution information but nonetheless are an extremely sensitive cellular method that allows us to rapidly identify small molecules that cause minute structural changes in αSN oligomers. This sensitivity is enhanced with our use of multiple, distinct FRET biosensors. Coupling the FRET response from the inter- and intra-protomeric biosensors provide further insight into each Hit’s MOA. The collective results of our FRET, cytotoxicity, pS^129^ pathology, smFRET and PrOF NMR, and in vitro oligomerization assay suggest that our hit compounds bind to and induce an increase in αSN oligomerization, biased toward non-toxic αSN oligomers where constituent monomers are in more extended conformations. That both DEM and RO are effective at low nM concentrations in cells (Fig. 8a), increase the formation of αSN oligomers (Fig. 9 and Supplementary Figs. 21 and 22) and slow or prevent growth of pre-formed fibrils in vitro (Figs. 10 and 11 and Supplementary Fig. 23), and reduce the presence of pS^129^-positive LMW oligomers in cells (Supplementary Fig. 18) may be some of the first evidence to suggest a targetable binding site that persists from oligomer to fibril.

Though we are far from providing definitive proof of persistent, robust targetable motifs, our pS^129^ data from both the mouse primary neuron model and SH-SY5Y model are also at least consistent with this possibility. Given the vastly different experimental platform of these experiments compared to our screening platform (e.g., mouse vs. human αSN in the primary neuron assay, spontaneous oligomerization vs. exogenous PFF treatment), that we saw an effect across systems is compelling. One possibility is that the compounds are directly binding to the PFF or oligomers in the PFF mixture, altering their growth and stability, or sterically altering accessibility to modifying enzymes. This would imply the existence of a persistent and robust motif targeting human αSN oligomers in HEK293 cells, SH-SY5Y cells (RO and DEM but not BAY for cytotoxicity), mouse αSN PFFs in primary neurons (DEM and BAY), and human αSN PFFs in differentiated SH-SY5Y cells (RO and DEM, and not BAY, reduce oligomers; RO, DEM, and BAY increase HMW assemblies).

In a recent study, the Volpicelli-Daley group detailed the nuanced in vivo pathological phenotypes associated with αSN oligomers and fibrils, demonstrating that, although both non-seeding-competent oligomers and PFF are capable of inducing neurotoxicity and synaptic deficits, only the latter PFF treatment resulted in PD-like staging of pathology and motor/behavioral phenotypes^105^. Although our cellular FRET biosensors monitor changes in oligomeric αSN, our hit compounds do display the capacity of disrupting both PFF-induced pathological phenotypes (Fig. 8 and Supplementary Figs. 16–18) and seeded αSN fibrillization (Fig. 11 and Supplementary Fig. 23). These results suggest that these hit compounds may be able to disrupt αSN PFF seeding and cell-to-cell spread, highlighting an exciting direction for future studies to evaluate our hits in more complex and physiologically relevant neuronal or animal models. Nevertheless, the ability of our drug discovery platform to identify hit compounds that can target a multitude of therapeutic MOAs further demonstrates the broad potential application of our cellular αSN oligomer FRET biosensors.

A second, indirect MOA is possible as well and may explain why BAY showed an effect in the HEK293 cells and the mouse model but not in SH-SY5Y cells. Small molecules that alter a wide range of auxiliary cellular processes (e.g., activating the unfolded protein response, the endoplasmic reticulum stress response, etc.) could indirectly alter oligomeric structures (HEK293 cells), reduce cell death (SH-SY5Y), reduce phosphorylation of Ser129 (mouse primary neurons), and alter the distribution of pS^129^ assemblies (differentiated SH-SY5Y cells) without ever binding to αSN. But, again, the inherent difficulty in assessing direct binding of small molecules to a heterogeneous array of structured and unstructured oligomers in cells makes a definitive assignment of MOA, direct or indirect, challenging. Despite this, it is possible that compounds that act indirectly may ultimately prove the most potent, as they do not face the hurdle of needing to alter a complex heterogeneous ensemble of oligomers.

The collection of experiments presented here do not definitively elucidate the hit compounds’ MOA for altering αSN oligomers, cytotoxicity, and pathology. Indeed, in our two distinct pS^129^ pathology models we observe model-dependent differences: BAY and DEM reduce total pS^129^ in primary mouse neurons (Fig. 8) and only RO reduces total pS^129^ in differentiated SH-SY5Y cells (Supplementary Fig. 18). These inconsistencies undoubtedly result from the multitude of differences between the two assays (mouse vs. human αSN PFF; primary mouse neuron vs. human neuronal cell line; treatment duration (12 days vs. 2 days) or detection methodology (immunofluorescence cytochemistry vs. immunoblots). Hit compound effects become even more intriguing with the significant reduction in monomer pS^129^ fraction (RO, BAY, and DEM) and LMW oligomer pS^129^ fraction (RO and DEM) with a relative increase in the fraction of HMW assemblies (Supplementary Fig. 18). These apparent increases in HMW species could be due to a number of things (e.g., changes in PFF cellular uptake; changes in PFF clearance; remodeling of PFF or αSN(m)). Furthermore, it is likely that these HMW assemblies are a complex mixture of toxic and non-toxic species whose acute properties can be difficult to delineate, especially in cellular experiments. The effect of Baicalin (control compound in Supplementary Fig. 18) in our SH-SY5Y pS^129^ assay highlights this challenge, as Baicalin is known to remodel αSN PFF into non-toxic amorphous aggregates, yet we still observe a strong HMW pS^129^ signal. RO has been previously shown to inhibit Aβ fibrillization through interaction with structural intermediates^106^ and may have a similar direct MOA with αSN. DEM (a tetracycline derivative) may share properties with other tetracycline-like molecules (i.e., doxycycline), which has been shown to induce off-pathway oligomerization of αSN^107^.

Nevertheless, the primary focus of this study is the demonstration of our HTS platform’s ability to identify small molecules capable of modulating αSN oligomer structure and associated pathological phenotypes. Our screening platform, predicated on FRET measurements that monitor alterations in αSN’s oligomeric state, has successfully identified compounds that shift the relative ratios of the various oligomeric populations (Figs. 3 and 9 and Supplementary Figs. 18, 21, and 22). The exact mechanism by which those alterations change αSN pathology, a subject of essential debate in the field, should be the subject of future work.

Cellular αSN FRET biosensors have been previously developed and used successfully as sensitive biomarkers to detect seeding-competent αSN assemblies in patient-derived biofluids^108^. These earlier biosensors have been focused on characterizing cell-to-cell seeding and fibrillar αSN. The biosensor systems described here can be similarly used and the FLT detection scheme may improve the overall sensitivity of these approaches. Comparisons between our biosensor system and those previously developed are planned for future studies. BiFC has also been used before as a means to monitor αSN oligomerization^42,44^. Direct protein–protein interaction and fluorophore maturation is required for BiFC fluorescence. However, a disadvantage of using these systems for small-molecule screening is that it requires the compounds to dissociate the αSN assemblies by disrupting both the BiFC tag and target protein–protein interaction. In each of these approaches, including ours, there is the concern regarding XFP-induced artifacts. To reduce potential false positive hit detection, XFP fusion proteins are used solely in our primary HTS and confirmatory counter screens and FRET dose-response experiments. All other cellular assays are performed with unlabeled WT αSN (no fusion constructs). Furthermore, for assays with XFP fusion constructs all experiments are repeated with control biosensors to rule out non-specific FRET contributions.

To conclude, our inter- and intra-protomeric FRET αSN biosensors provide a new platform for evaluating αSN conformation and oligomerization. Extensions of our screening platform moving forward will focus on finding increasingly potent and central nervous system-penetrant small molecules and will be designed to answer many open questions. For example, screening in a variety of neuronal cell lines can address the ongoing question of whether variations in cellular milieu alters oligomeric structures (Are there common or disparate hits?), and screening against known familial mutants could clarify whether those also associate with differences in the structures of these toxic assemblies.

## Methods

### Molecular biology

To generate the αSN FRET constructs, αSN cDNA was cloned into an EGFP-linker-TagRFP plasmid (linker contains 32 amino acids, GFP-32AA-RFP) that was previously characterized^50^. QuikChange mutagenesis (Agilent Technologies, Santa Clara, CA) was performed to monomerize the GFP via A206K mutation and to produce the donor GFP-αSN construct^109^. Subsequent QuikChange mutagenesis was used to remove the 32 amino acid linker and stop codon after αSN (creating GFP-αSN-RFP construct) as well as to excise the GFP (creating unlabeled αSN and αSN-RFP constructs). The αSN A53T mutation was introduced into all four plasmids. Primers for all QuikChange mutagenesis are detailed in Supplementary Table 3. All biosensor plasmid constructs were sequenced for confirmation (ACGT, Wheeling, IL).

### Cell culture

HEK293 and SH-SY5Y cells (ATCC) were cultured in phenol red-free Dulbecco’s Modified Eagle Medium (Gibco) supplemented with 2 mM L-Glutamine (Invitrogen), heat-inactivated 10% fetal bovine serum (Gibco), 100 U/mL penicillin, and 100 μg/mL streptomycin (Gibco). Cell cultures were maintained in an incubator with 5% CO_2_ (Forma Series II Water Jacket CO_2_ Incubator, Thermo Scientific) at 37 °C. The inter-protomeric (oligomer) and intra-protomeric (oligomer and conformation) αSN FRET biosensors were generated by transiently transfecting HEK293 cells using Lipofectamine 3000 (Invitrogen) with GFP-αSN and αSN-RFP (1:8 DNA plasmid concentration ratio) or GFP-αSN-RFP plasmid, respectively. The effectiveness of HEK293 cells transfected with FRET constructs as an HTS platform has been demonstrated in our previous work^46–52,66^.

### LOPAC library and liquid handling

The LOPAC (Sigma-Aldrich) contains 1280 compounds that span marketed drugs, failed development candidates, and naturally occurring compounds that have well-characterized activities with widely described biological effects. The library is originally formatted in 96-well mother plates and dispensed across four 384-well and/or one 1536-well flat, black-bottom polypropylene plates at 10 μM final concentration/well (50 μL final volume for 384-well; 10 μL for 1536 wells) using an automated Echo acoustic liquid dispenser from Labcyte (Sunnyvale, CA, USA). DMSO (matching %v/v) was loaded as in-plate no-compound negative controls to make a total of 960 wells (384-well plates) or 256 wells (1536-well plate). The plates were sealed and stored at −20 °C until use.

Two days prior to screening, HEK293 cells were transfected using Lipofectamine 3000 with GFP-αSN/αSN-RFP (αSN oligomer FRET biosensor) or GFP-αSN-RFP (αSN monomer conformation FRET biosensor) in 15× 100 mm plates (6 × 10^6^ cells/plate). On each day of screening, the compound plates were equilibrated to room temperature (RT; 25 °C). The cells were harvested from the 100-mm plates by incubating with TrypLE (Invitrogen) for 2 min, washed three times in PBS by centrifugation at 200 × *g*, and filtered using 70-μm cell strainers (BD Falcon). Cell viability prior to screen was assessed using a trypan blue assay and confirmed to be >80%. Cells were diluted to 1.5E6 cells/mL. Expression of GFP-αSN and the αSN biosensors were confirmed by fluorescence microscopy prior to each screen (EvosFL-Auto microscope, Thermo Fisher Scientific, USA). During screening, cells (50 μL/well for 384-well; 10 μL/well for 1536-well) were dispensed by a Multidrop Combi liquid dispenser (Thermo, Pittsburg, PA, USA) into the assay plates containing the compounds and allowed to incubate at RT for 90 min before recording with the FLT-PR or SUPR (both instruments manufactured by Fluorescence Innovations Inc., both owned and provided by Photonic Pharma LLC), as described previously^46^. Reproducible FRET hits for retesting were purchased from Tocris (Minneapolis, MN, USA), Sigma (St. Louis, MO, USA), or Invitrogen (Carlsbad, CA, USA), depending on availability.

### HTS and FLT data analysis

As described previously^46,47,50^, time-resolved fluorescence waveforms for each well were fit with single-exponential decays using the least-squares minimization global analysis software to give donor–acceptor lifetime (*τ*_DA_) and donor-only lifetime (*τ*_D_). FRET efficiency (*E*) was then calculated based on Eq. 1.

1 $$E = 1 - \left( {\frac{{\tau _{{\mathrm{DA}}}}}{{\tau _{\mathrm{D}}}}} \right)$$

Assay quality was determined using control EGCG and hit compound DEM as positive controls and DMSO as a negative control and calculated based on Eq. 2^110^,

2 $$Z^\prime = 1 - \frac{{3(\sigma _{\mathrm{p}} + \sigma _{\mathrm{n}})}}{{|\mu _{\mathrm{p}} - \mu _{\mathrm{n}}|}}$$

where *σ*_p_ and *σ*_n_ are the SDs of the observed *τ*_DA_ values and *µ*_p_ and *µ*_n_ are the mean *τ*_DA_ values of the positive and negative controls.

Interfering compounds were flagged as potential false positives by a set of stringent FC filters based on spectral analysis of each well from the LOPAC screen^45,46^. After removal of FCs, a histogram of the FLT distribution from all compounds in the screen was plotted and fit to a Gaussian curve to obtain the mean (*µ*) and SD (*σ*). A hit was defined as a compound that attenuated the FLT efficiency by >5*σ* relative to the mean *µ* (Supplementary Fig. 12).

Initial biosensor characterization was performed via a titration of donor and acceptor DNA into HEK293 cells. Cells were transiently transfected and incubated for 48 h, harvested, washed, and dispensed with 5 replicates for FLT characterization in the FLT-PR. Figure 1a illustrates a robust donor-only (1:0) FLT that undergoes increasing FLT attenuation with increased acceptor ratio through D:A of 1:12. There is a plateau in FLT reduction that correlates with overall donor signal to noise. Figure 1b presents the corresponding FRET for each system determined via Eq. 1, where there is significant increase in basal FRET up to D:A = 1:8.

### FLT dose-response assay

Compounds were tested in a FLT dose-response assay. The compound was dissolved to 10 mM in DMSO, then serially diluted in 96-well mother plates. Each hit was re-screened for FLT dose response across eight concentrations (1 nM to 50 μM). Compounds (0.5 μL) were transferred from mother plates into assay plates using a Mosquito HV liquid handler (TTP Labtech Ltd, UK). The preparations for αSN FRET biosensors were carried out as above.

### Spectral unmixing experiment and spectral data analysis

For each biosensor system, the fluorescence emission spectrum was recorded and analyzed as described previously^66^. In brief, biosensor cells are harvested and prepared as for FLT-PR experiments and dispensed in either 1536- or 384-well plates (Greiner Bio-One) at a cell density of 1e6 cells/mL (5 or 50 μL/well, respectively). Each sample was excited using a 473-nm, continuous-wave laser and fluorescence emission spectra recorded across the entire visible spectrum with the 500–700-nm range used in spectral fitting.

The observed spectrum *F*(*λ*) was fitted by least-squares minimization to a linear combination of component spectra

3 $$F_{{\rm{fit}}}\left( \lambda \right) = aF_{\rm{D}}\left( \lambda \right) + bF_{\rm{A}}\left( \lambda \right) + cF_{\rm{C}}\left( \lambda \right) + dF_{\rm{W}}\left( \lambda \right)$$

where D is donor, A is acceptor, C is cell autofluorescence background, and W is water Raman scattering and a–d are the coefficients determined from the fit to a series of basis functions defined for each component. The fitted spectrum is then determined using least-squares minimization with Matlab (MathWorks)^66^.

### Flow cytometry

After biosensor cells were harvested and prepared for FLT-PR experiments, an aliquot of cells (~1e6 cells in total) was collected and processed via flow cytometry. Biosensor-expressing cells were analyzed with a BD Accuri C6 flow cytometer. Initial gating was defined with untransfected and single GFP- or RFP-transfected cells to establish baseline for the co-transfected biosensor cells. Cell analysis was complete after 2e5 cellular events were recorded.

### Cell cytotoxicity assay

Cell cytotoxicity was measured using the CytoTox-Glo (Promega Corporation) Luminescence Assay Kit. SH-SY5Y human neuroblastoma cells were plated at a density of 5 × 10^6^ cells/plate in 100 mm plate (Corning) and transfected with unlabeled WT αSN or equivalent vector-only control for 24 h. The transfected cells were then plated at a density of 5000 cells/well in white solid 96-well plate (Corning) with a total volume of 100 μL, followed by treatment with hit compounds spanning a range of concentrations (0.1 nM to 100 μM), as well as DMSO-only controls, for another 48 h. After incubation, 50 μL of CytoTox-Glo Cytotoxicity Assay Reagent was added to all wells followed by mixing by orbital shaking and incubation in the dark for 15 min at RT. Luminescence readings were measured using a Cytation3 Cell Imaging Multi-Mode Reader luminometer (BioTek). After the first read, 50 μL of Lysis Reagent was added, incubated for 15 min at RT, and a second read performed. Total viable cellular luminance was determined by the difference between the first and second luminescence signal and total cellular viability is determined by normalizing to the no-treatment samples.

### Primary hippocampal neuron cultures and fibril transduction

Primary neuronal cultures were prepared from CD1 embryos on E16–18, as previously described^58^. Primary mouse neuron protocol was approved by the University of Pennsylvania Institutional Animal Care and Use Committee. Tissue culture plates and coverslips were coated with Poly-d-lysine (Sigma) before addition of cells. Neurons were plated in 96-well plates (60,000 cells/cm^2^). Cultures were maintained in Neurobasal medium supplemented with B27 and Glutamax (all from Invitrogen).

PFF treatment was performed at 7 DIV and cultures were incubated for a further 12 days prior to fixation and analysis. Briefly, stock αSN PFFs were diluted in sterile DPBS (without Ca^2+^/Mg^2+^; Corning)—8 μL of aSyn + 392 μL of DPBS into 1.5 mL Eppendorf tubes—and sonicated with a bath sonicator (Bioruptor Plus, Diagenode) for 10 cycles at high power (30 s on, 30 s off, at 10 °C). Sonicated PFFs were then further diluted to the indicated final concentration in neuronal media and added to cultures. PFF concentrations are expressed as the total equivalent αSN monomer content in the preparation. Cultures were treated with 200 nM PFFs unless otherwise noted. Compounds were initially diluted in neuronal media before being added to the PFF neuronal media solution at a final concentration of 1–10 μM.

### PFF and compound treatment in differentiated SH-SY5Y cells

SH-SY5Y cells were differentiated using reduced serum (3%) media supplemented with 10 μM retinoic acid for 3 days and then changed to serum-free media supplemented with 10 ng/mL brain-derived neurotrophic factor for the remainder of the experiment. Stock αSN PFFs were diluted and sonicated as described previously. SH-SY5Y cultures were then treated with 200 nM PFF and PBS, DMSO, 1 μM drug compound (RO, BAY, or DEM), or 50 μM Baicalin. After 48 h, SH-SY5Y were washed with PBS and harvested in cold lysis buffer with protease and phosphatase inhibitors, as previously described. Approximately 15 μg of total protein was loaded onto a 4–20% Criterion TGX midi gel and probed using Syn1 (BD Biosciences, #610787; 1° 1:5000; 2° 1:10K) and pS^129^ aSyn (abcam, ab59264; 1° 1:1000; 2° 1:5000) and a-tubulin (abcam, ab4074; 1° 1:1000; 2° 1:10K)

### Immunocytochemistry and antibodies

Cultured neurons were fixed by replacing media with warm PFA (4% in PBS containing 4% sucrose) for 15 min at RT. Fixed neurons were washed three times with PBS and then blocked (3% bovine serum albumin, 3% fetal bovine serum in PBS) for 1 h at RT. Cells were then incubated in primary antibodies diluted in blocking buffer for 1 h at RT. Primary antibodies used in this study were: p-S129 αSN (pSyn; CNDR mouse monoclonal IgG_2a_ 81A; 1:3000), NeuN (Millipore A60; 1:3000), MAP2 (CNDR rabbit monoclonal 17028: 1:3000). This was followed by washing with PBS three times and incubating for 1 h at RT in blocking buffer containing Alexa-Fluor-conjugated isotype-specific secondary antibodies (Thermo Fisher). Cells were washed three times with PBS and 4,6-diamidino-2-phenylindole (0.4 µg/mL in PBS) was added to visualize cell nuclei. Stained cells were imaged using an InCell 2200 (GE Healthcare Life Sciences). Images from 9 fields were collected from each well with a ×20 objective. Image analysis and quantification was performed using the InCell Toolbox Analyzer software (GE Healthcare Life Sciences). The total integrated area for 81a immunopositivity was quantified for each sample and then normalized (divided) by the number of NeuN-positive objects in the fields examined. Our approach did not distinguish between ankyrin/puncta structures vs. more elongated pathologies that have also been described in this model system.

### Western blot analysis for biosensor expression

To test the expression of αSN FRET biosensors, HEK293 cells were plated in a 6-well plate at a density of 1 × 10^6^ cells/well and transfected with GFP-αSN/αSN-RFP plasmids. Cells were lysed for 30 min on ice with radioimmunoprecipitation assay (RIPA) lysis buffer (Pierce RIPA buffer, Thermo Fisher Scientific) containing 1% protease inhibitor (Clontech, Mountain View, CA) and 1% phosphatase inhibitors (Millipore Sigma), and centrifuged at 15,000 × *g* at 4 °C for 15 min. The total protein concentration of lysates was determined by bicinchoninic acid (BCA) assay (Pierce), and equal amounts of total protein (60 μg) were mixed with 4× Bio-Rad sample buffer and loaded onto 4–15% Tris-glycine SDS-PAGE gels (Bio-Rad, Hercules, CA). Proteins were transferred to supported nitrocellulose membrane and probed using Syn1 and pS^129^ aSyn antibodies against αSN. Blots were imaged on using a ChemiDoc MP imager and analyzed using Image Lab (Bio-Rad, Hercules, CA). All blots used for quantification derived from the same experiment and were processed in parallel.

### Triton-X 100 soluble/insoluble fractions

HEK cells were transiently transfected with empty vector or GFP-αSN/αSN-RFP as previously described. Cells were treated with either PBS or PFF seeds (4 μg/mL) 24 h after transfection. After 24 h of treatment, cells were harvested in cold lysis buffer (50 mM Tris/HCl pH 7.4, 150 mM NaCl, 5 mM EDTA pH 8.0) with protease and phosphatase inhibitors and 1% Triton-X 100 and incubated on ice for 30 min. The lysates were then centrifuged at 25,000 × *g* for 60 min at 4 °C. The supernatant was collected as the Triton-X 100 soluble fraction and the pellet was resuspended in lysis buffer with 2% SDS and sonicated for 10 s and collected as the Triton-X 100 insoluble fraction. Both fractions were boiled and then a BCA protein assay was used to quantify the protein concentration of the samples. Approximately 12 μg of total protein was loaded onto a 4–20% mini-Protean TGX gel (Bio-Rad, Hercules, CA), and blots were evaluated as described previously.

### Immunoprecipitation

GFP-Trap magnetic agarose from Chromotek (Munich, Germany) were used for IP per the manufacturer’s instructions. HEK cells were transiently transfected as previously described with αSyn biosensor plasmids and with appropriate controls [HEK control cells, GFP:αSN, GFP:αSN-RFP, GFP-αSN:RFP, GFP-αSN: αSN, GFP-αSN: αSN-RFP (D:A 1:8), and GFP-αSN: αSN-RFP (D:A 1:12)]. Cells were harvested in ice-cold native lysis buffer 48 h after transfection and agitated for 60 min at 4 °C. The cellular lysates were centrifuged 12,000 RPM for 20 min. The supernatant was collected used for IP. Diluted supernatant was added to the equilibrated GFP-Trap magnetic agarose and rotated at 4 °C overnight. Proteins were eluted with 2× SDS Laemmli sample buffer and analyzed using SDS-PAGE.

### Protein purification and labeling

αSN was purified from *Escherichia coli* cells as described previously^111^. Briefly, αSN in a T7–7 plasmid was transformed into BL21(DE3) cells and grown overnight in 10 mL LB media with 100 μg/mL ampicillin; the overnight culture was used to inoculate 500 mL LB/100 μg/mL ampicillin. At OD_600_ = 0.5–0.6, protein expression was induced by the addition of 320 μM IPTG for 4 h at 37 °C. The cells were pelleted and resuspended in 25 mL lysis buffer (40 mM NaOH, 20 mM Tris pH 8.0, 1 mM EDTA, 1 mM phenylmethanesulfonylfluoride (PMSF), 0.1% Triton X-100) and a complete protease inhibitor cocktail tablet. The lysate was then flash-frozen in liquid nitrogen and stored at −80 °C until use. The thawed lysate was treated with 200 U of DNase supplemented with 10 mM MgCl_2_ and 10 mM CaCl_2_ and incubated for 1 h at 37 °C shaking. In all, 10 mM EDTA was added prior to centrifugation at 16,900 × *g* for 15 min to remove cellular debris. Two ammonium sulfate cuts (0.116 and 0.244 g/mL) are added to the supernatant, resulting in αSN precipitating in the second step. The pellet was resolubilized in Buffer A (25 mM Tris pH 8.0, 20 mM NaCl, 1 mM EDTA) with 1 mM PMSF and dialyzed against Buffer A to remove ammonium sulfate. The supernatant was filtered through a 0.22-μm syringe filter and then loaded to an anion exchange column (GE HiTrap Q HP, 5 ml) and eluted with a gradient to 1 M NaCl. αSN elutes at approximately 300 mM NaCl. Fractions containing αSN were pooled, concentrated, and buffer exchanged into Buffer C (25 mM Tris pH 8, 100 mM NaCl, 1 mM EDTA, 0.5 mM TCEP) using Amicon Ultra concentrators (3000 Da MW cutoff). Concentrated samples were once again syringe filtered before loading on a size exclusion column (GE HiLoad 16/600 Superdex75) and eluted at 0.5 mL/min. Fractions containing αSN were again pooled and concentrated, then stored at −80 °C.

For site-specific labeling, cysteines were introduced at residues 9 and 72 or 54 and 130. αSN was incubated with 1 mM dithiothreitol for 30 min and then buffer exchanged into labeling buffer (20 mM Tris pH 7.4, 50 mM NaCl, and 6 M guanidine HCl). Protein was incubated with the donor fluorophore Alexa Fluor 488 maleimide at a ratio of protein:dye of 1:0.5 for 2 h at RT with stirring. Then, 4× molar excess of acceptor fluorophore Alexa Fluor 594 maleimide was added and incubated overnight at 4 °C. Excess dye was removed by buffer exchanging into 20 mM Tris (pH 7.4) and 50 mM NaCl using Amicon concentrators and two tandem HiTrap Desalting Columns. Labeled proteins were aliquoted, snap frozen, and stored at −80 °C until use.

### Pre-formed fibrils

Bulk PFFs were prepared following the protocol developed by Virginia Lee’s group^58^. Briefly, aliquots of recombinant αSN(m) were rapidly thawed and spun at 25,000 × *g* for 30 min at 4 °C. For bulk PFF production, approximately 500 μL of 5 mg/mL monomeric protein was added to a 2-mL Eppendorf tube and placed on a thermomixer-C (Eppendorf, USA) shaker and incubated at 37 °C and 1000 RPM for up to 144 h. Every 6 h, 5 μL aliquots were removed and assayed for ThT fluorescence until a plateau in aggregation was observed and the tube was opaque. Twenty microliters of aliquots of 5 mg/mL PFF were then flash frozen and stored at −80 °C.

### αSN oligomerization and cross-linking assay

In vitro recombinant αSN oligomerization assay was adapted from the methods described by Otzen et al.^90^. In brief, aliquots of purified recombinant αSN were quick-thawed and centrifuged at 25,000 × *g* for 30 min at 4 °C. The top 95% of supernatant was removed and pooled. Concentration was determined using UV280 absorbance on a Spectromax-i3x (Molecular Devices) and the protein was diluted to a final concentration of 5 mg/mL (~350 μM). Aliquots of 150 μL were dispensed into 2-mL low-protein-binding centrifuge tubes and compounds were added to each tube for a final concentration of 70 μM (1:5 compound:protein ratio). Samples were then incubated under continuous shaking (900 RPM) at 37 °C for 12 h (ThermoMixer-C, Eppendorf, Hauppauge, NY).

After oligomerization, a 50-μL aliquot was removed and cross-linked following the protocol developed by Jensen et al.^91^. In brief, oligomer aliquot was incubated with 1.6% PFA in PBS for 1 h at ambient temperature. Cross-linking was quenched with tenfold excess Tris (pH 7.4) and equal volume was prepared in 4× Laemmli loading buffer (BioRad) for subsequent SDS-PAGE, transfer, and immunoblot analysis as previously detailed above.

A 10-μL aliquot from each sample was taken and diluted with 290 μL ThT working solution (100 μM ThT in PBS) and dispensed into 3 wells of a 384-well, black opaque plate. Additional samples of PBS, αSN(m), and αSN PFF were used as controls. Plates were incubated at ambient temperature for 10 min and then fluorescence read at 450ex/490em on a Spectromax-i3x (Molecular Devices).

### ThT aggregation assays: non-seeded, seeded, and cellular homogenate

ThT (Sigma, product no. T3516) was dissolved in PBS buffer and was filtered through a 0.2-µm syringe filter to make a stock solution of 2.5 mM. Working ThT concentration was 20 µM. PFF seeds were produced via constant agitation of 1000 RPM at 37 °C (ThermoMixer-C, Eppendorf, Hauppauge, NY) of 500 µL of 350 µM αSN in PBS in a 2 mL tube for 5 days. PFFs were aliquoted and flash frozen and stored at −80 °C until further use. PFF seeds were rapidly thawed, diluted to 35 µM in PBS, and sonicated 120 pulses at 50% using a probe-tip sonicator following the established protocols^58^. Master mix samples for each of the different ThT assays were prepared and dispensed in triplicate as 50 µL in 384-well flat, black-bottom polypropylene plates (PN 781209, Greiner Bio-One) and sealed with fluorescently transparent film. Conditions for non-seeded, seeded, and cell homogenate assays varied slightly.

#### Non-seeded ThT

Non-seeded ThT master mix was prepared in cell culture PBS with 140 µM (2 mg/mL) αSN(m) and dispensed into compound plates at 70 µM final compound concentration (1:2 compound:protein ratio).

#### Seeded ThT

Seeded ThT experiments were performed at a lower monomer concentration to preclude spontaneous αSN aggregation during the time course of the experiment. For both seeded and cell homogenate experiments, sample were prepared in PBS + 20 µM ThT with or without 15 µM αSN(m) +/− 5% PFF (0.75 µM). Additional control samples of 15 µM PFF and 0.75 µM PFF were also prepared. All empty wells were filled with PBS for a thermal sink and to help prevent evaporation. Assay plates were prepared as pre-cellular FRET dose response above. Dose response was done across 8 concentrations (10 nM to 50 µM). The assay was run at 37 °C with mild shaking (200 RPM) in a Cytation3 plate reader. The ThT fluorescence was monitored with excitation filter of 450 nm and emission filter of 490 nm. Readings were acquired every 20 min for a minimum of 24 h. Seeded ThT aggregation IC_50_ values were determined by first normalizing all ThT curves to the maximal signal at plateau for the αSN + 5% PFF + DMSO samples. The normalized fluorescence at each concentration was fit to the Hill equation to resolve an effective IC_50_ of seeded aggregation inhibition.

#### Cell homogenate ThT

Cell homogenate ThT experiments were performed with inter-protomeric αSN biosensors expressed in HEK293 cells as described above. Cells were homogenized in native lysis buffer with proteasome inhibitor. After lysis, cells were centrifuged at 16,000 × *g* for 1 min and total protein concentration of the supernatant was quantified via BCA analysis. Cell lysates from the three different sets of plasmids, along with an untransfected HEK293 control, were combined with PBS and ThT (20 µM) to reach a 5% lysate seeded solution. A second set of test samples were combined with PBS, ThT, and 15 µM αSN(m). PBS only was used as background, with 5% PFF or αSyn(m) as negative control and 5% PFF with αSyn(m) as positive control. Samples were plated in triplicate in 384-well flat, black-bottom polypropylene plates (PN 781209, Greiner Bio-One) and sealed with fluorescently transparent film. ThT fluorescence was monitored using a Cytation3 (excitation: 450 nm, emission: 490 nm) under continuous shaking with reads every 10 min for 48 h.

### smFRET measurements

Single-molecule FRET measurements were carried out using ~30 pM of labeled αSN in 20 mM Tris pH 7.4 and 50 mM NaCl in 8-chambered Nunc coverslips (ThermoFisher) passivated with poly(ethylene glycol) poly(L-lysine) to reduce protein adsorption to the chambers. Measurements were made on a MicroTime 200 time-resolved confocal microscope (Picoquant) as described previously^112^. Laser power was adjusted to ~30 μW before sample illumination. Fluorescence emission was collected through the objective and passed through a 150-μm diameter pinhole. Photons were separated by an HQ585LP dichroic in combination with ET525/50M and HQ600LP filters and detected by avalanche photodiodes. Photon traces were collected in 1-ms time bins for 1 h. A cutoff of 25 counts/ms was applied to discriminate between bursts arising from fluorescently labeled protein and background noise. The FRET efficiency (*E*_eff_) was calculated using the SymphoTime 64 software. SmFRET histograms were fit with Gaussian distributions to determine the peak *E*_eff_ values. Each measurement was carried out three times and mean *E*_eff_ values are the average and SD of these measurements (Supplementary Table 2). Alignment of instrument and analysis were verified using smFRET reference standards consisting of 10, 14, and 18 base pair dsDNA labeled with Alexa 488 and Alex 594 with their 5’ and 3’ ends.

### Protein-observed fluorine NMR (PrOF NMR)

Experiments were run on a Bruker 600-MHz Avance NEO (6001), equipped with a 5 mm triple resonance cryoprobe. 3FY-αSN was diluted in PBS, pH = 7.4 buffer by the addition of D_2_O and 0.1% trifluoroacetic acid (TFA) to final concentrations of 5 and 0.07%, respectively, in a total NMR sample volume of 150 µL. Two one-dimensional ^19^F NMR spectra were taken of the protein sample at an O1P of −75 ppm, NS = 16, D1 = 1 s, and AQ = 0.5 s (TFA reference set to −75.25 ppm) and an O1P of −136 ppm, NS = 3072, D1 = 0.6 s, and AQ = 0.05 s (protein resonances). Ligand stock solutions of 1–10 mM were prepared in DMSO and titrated into the protein solutions (20 μM). As a control experiment, in a separate NMR tube, DMSO was added to a final concentration of 0.5, 0.6, 0.8, 1.5, and 2.5% to control for any solvent-induced chemical shifts. For each concentration, a TFA reference spectrum and a protein 1D-^19^F NMR spectrum were acquired.

### Statistical analysis

Data are shown as mean ± SD unless stated otherwise. Statistical analysis for FLT and FRET experiments were conducted by an unpaired Student’s *t* test using GraphPad to determine statistical significance for all experiments. Values of *p* value <0.05 were considered statistically significant.

Outcome measures for PFF-induced neuron pathology (DxA 81A/NeuN ratio) were compared between experimental conditions using linear mixed-effects models, with fixed effects for condition and random effects for experiment, well by condition, and experiment by well by condition, to account for within-well correlation, variation by experiment, and variation of conditions across experiments. Outcome measures were log-transformed for analysis for approximate normality and homogeneity of variance across conditions and experiments. Estimated differences between conditions, along with their 95% confidence intervals, were exponentiated to report as ratios of the outcome measures between conditions. Analyses were conducted using R^113^ version 3.6.1, including the packages lme4^114^ version 1.1-21 and lmerTest^115^ version 3.1-1.

### Reporting summary

Further information on research design is available in the Nature Research Reporting Summary linked to this article.

## Supplementary information

Reporting Summary

Supplementary Information

## Data Availability

The datasets generated and/or analyzed during the current study are available from the corresponding author on reasonable request.

## Code availability

Analyses for the FLT/SUPR HTS data were performed with custom scripts for waveform fitting and spectral unmixing (Matlab 2019a) and Hit identification (Excel 2016). Analyses for the primary neuron pathology assay were conducted using R (version 3.6.1), including the packages lme4^114^ version 1.1–21 and lmerTest^115^ version 3.1-1. Scripts are available from the corresponding author on reasonable request.
