# Supplementary material for: Potent inhibitors of toxic alpha-synuclein identified via cellular time-resolved FRET biosensors
Source: NPJ Parkinsons Dis. 2021 Jun 28;7:52. doi: 10.1038/s41531-021-00195-6 (PMC8238948; doi:10.1038/s41531-021-00195-6)
Supplement: Supplementary file 1 — Reporting Summary [file 41531_2021_195_MOESM1_ESM.pdf]

nature research

Corresponding author(s): Jonathan N. Sachs

Last updated by author(s): May 14, 2021

# Reporting Summary

Nature Research wishes to improve the reproducibility of the work that we publish. This form provides structure for consistency and transparency in reporting. For further information on Nature Research policies, see our Editorial Policies and the Editorial Policy Checklist.

## Statistics

For all statistical analyses, confirm that the following items are present in the figure legend, table legend, main text, or Methods section.

| n/a | Confirmed | |
|---|---|---|
| ☐ | ☒ | The exact sample size ($n$) for each experimental group/condition, given as a discrete number and unit of measurement |
| ☐ | ☒ | A statement on whether measurements were taken from distinct samples or whether the same sample was measured repeatedly |
| ☐ | ☒ | The statistical test(s) used AND whether they are one- or two-sided<br>*Only common tests should be described solely by name; describe more complex techniques in the Methods section.* |
| ☐ | ☒ | A description of all covariates tested |
| ☐ | ☒ | A description of any assumptions or corrections, such as tests of normality and adjustment for multiple comparisons |
| ☐ | ☒ | A full description of the statistical parameters including central tendency (e.g. means) or other basic estimates (e.g. regression coefficient) AND variation (e.g. standard deviation) or associated estimates of uncertainty (e.g. confidence intervals) |
| ☒ | ☐ | For null hypothesis testing, the test statistic (e.g. $F$, $t$, $r$) with confidence intervals, effect sizes, degrees of freedom and $P$ value noted<br>*Give P values as exact values whenever suitable.* |
| ☒ | ☐ | For Bayesian analysis, information on the choice of priors and Markov chain Monte Carlo settings |
| ☒ | ☐ | For hierarchical and complex designs, identification of the appropriate level for tests and full reporting of outcomes |
| ☒ | ☐ | Estimates of effect sizes (e.g. Cohen's $d$, Pearson's $r$), indicating how they were calculated |

*Our web collection on statistics for biologists contains articles on many of the points above.*

## Software and code

Policy information about availability of computer code

| | |
|---|---|
| Data collection | Fluorescence lifetime and spectral measurements were made using custom software developed and supported by Photonic Pharma LLC. Western blot detection and densitometry was performed with ImageLab (BioRad). Thioflavin-T fluorescence and Cytotox-GLO luminescence data was collected on a Cytation3 with BioTek Gen5. Flow Cytometry data was collected on BD-AccuriC6. NMR spectra was collected with TopSpin. |
| Data analysis | All data analysis was done using Microsoft Excel365 and graphs generated with GraphPad Prism 8.0. Cell Micrograph images were processed with GIMP. |

For manuscripts utilizing custom algorithms or software that are central to the research but not yet described in published literature, software must be made available to editors and reviewers. We strongly encourage code deposition in a community repository (e.g. GitHub). See the Nature Research guidelines for submitting code & software for further information.

## Data

Policy information about availability of data

All manuscripts must include a data availability statement. This statement should provide the following information, where applicable:

- Accession codes, unique identifiers, or web links for publicly available datasets
- A list of figures that have associated raw data
- A description of any restrictions on data availability

The datasets generated and/or analyzed during the current study and corresponding analysis scripts are available from the corresponding author on reasonable request.

# Field-specific reporting

Please select the one below that is the best fit for your research. If you are not sure, read the appropriate sections before making your selection.

☒ Life sciences ☐ Behavioural & social sciences ☐ Ecological, evolutionary & environmental sciences

For a reference copy of the document with all sections, see nature.com/documents/nr-reporting-summary-flat.pdf

# Life sciences study design

All studies must disclose on these points even when the disclosure is negative.

| | |
|---|---|
| Sample size | Sample size was not predetermined for this study. Individual experiments were performed with replicate measures of at least n=3. |
| Data exclusions | No data was excluded from the study. |
| Replication | Experiments were performed in triplicate to demonstrate reproducibility. Prior to biosensor screens, FRET level was established and coefficient of variance determined to ensure sufficient signal window for the experiment. |
| Randomization | No randomization was performed in this study. All hit compounds were determined from predefined drug plates and the number of hits evaluated were small enough to all be evaluated when available. |
| Blinding | Investigators were blinded to the drug plate layout for the initial high-throughput screens. Collaborating investigators were blind to the actual hit compound name evaluated (being given only an arbitrary labeling scheme). |

# Reporting for specific materials, systems and methods

We require information from authors about some types of materials, experimental systems and methods used in many studies. Here, indicate whether each material, system or method listed is relevant to your study. If you are not sure if a list item applies to your research, read the appropriate section before selecting a response.

## Materials & experimental systems

| n/a | Involved in the study | |
|---|---|---|
| ☐ | ☒ | Antibodies |
| ☐ | ☒ | Eukaryotic cell lines |
| ☒ | ☐ | Palaeontology and archaeology |
| ☒ | ☐ | Animals and other organisms |
| ☒ | ☐ | Human research participants |
| ☒ | ☐ | Clinical data |
| ☒ | ☐ | Dual use research of concern |

## Methods

| n/a | Involved in the study | |
|---|---|---|
| ☒ | ☐ | ChIP-seq |
| ☐ | ☒ | Flow cytometry |
| ☒ | ☐ | MRI-based neuroimaging |

## Antibodies

| | |
|---|---|
| Antibodies used | Syn-1, BD Biosciences, Catalog No. 610787;<br>LB509, Santa Cruz Biotechnology, sc-58480;<br>pSyn129; Abcam, ab184674; CNDR mouse monoclonal IgG2a 81A;<br>NeuN, Millipore, MAB377; Clone A60;<br>MAP2, CDNR, 17028: |
| Validation | *Describe the validation of each primary antibody for the species and application, noting any validation statements on the manufacturer's website, relevant citations, antibody profiles in online databases, or data provided in the manuscript.* |

## Eukaryotic cell lines

Policy information about cell lines

| | |
|---|---|
| Cell line source(s) | HEK293T (ATCC); SH-SY5Y (ATCC) |
| Authentication | Cell lines were not authenticated after initial receipt from ATCC |
| Mycoplasma contamination | Cell lines were not tested for mycoplasma contamination |
| Commonly misidentified lines (See ICLAC register) | *Name any commonly misidentified cell lines used in the study and provide a rationale for their use.* |

## Flow Cytometry

### Plots

Confirm that:

☒ The axis labels state the marker and fluorochrome used (e.g. CD4-FITC).

☒ The axis scales are clearly visible. Include numbers along axes only for bottom left plot of group (a 'group' is an analysis of identical markers).

☒ All plots are contour plots with outliers or pseudocolor plots.

☒ A numerical value for number of cells or percentage (with statistics) is provided.

### Methodology

| | |
|---|---|
| Sample preparation | HEK293 cells were lifted with TrypLE, washed in PBS, and resuspended @ 2E6 cells/mL in PBS |
| Instrument | BD Accuri™ C6, BD Biosciences |
| Software | Accuri™ C6 Software |
| Cell population abundance | *Describe the abundance of the relevant cell populations within post-sort fractions, providing details on the purity of the samples and how it was determined.* |
| Gating strategy | Gating on quadrants of donor (GFP), acceptor (RFP); and co-expressing (double positive) were determined based on untransfected and singly GFP/RFP transfected cells. |

☒ Tick this box to confirm that a figure exemplifying the gating strategy is provided in the Supplementary Information.
