# Supplementary material for: Potent inhibitors of toxic alpha-synuclein identified via cellular time-resolved FRET biosensors
Source: NPJ Parkinsons Dis. 2021 Jun 28;7:52. doi: 10.1038/s41531-021-00195-6 (PMC8238948; doi:10.1038/s41531-021-00195-6)
Supplement: Supplementary file 2 — Supplementary Information [file 41531_2021_195_MOESM2_ESM.pdf]

Potent inhibitors of toxic alpha-synuclein identified via cellular time-resolved FRET biosensors.

Supplemental Figures

Anthony R. Braun[a], Elly E. Liao[a], Mian Horvath[b], Prakriti Kalra[d], Karen Acosta[h], Malaney C. Young[a], Noah Nathan Kochen[a], Chih Hung Lo[a], Roland Brown[c], Michael D. Evans[c], William C. K. Pomerantz[d], Elizabeth Rhoades[g,h], Kelvin Luk[b], Razvan L. Cornea[e, f], David D. Thomas[e, f], and Jonathan N. Sachs[a*].

[a] Dept. of Biomedical Engineering, University of Minnesota, Minneapolis, MN 55455

[b] Dept. of Pathology and Laboratory Medicine, University of Pennsylvania, Philadelphia, PA 19104

[c] Clinical and Translational Science Institute, University of Minnesota, Minneapolis, MN 55455

[d]Dept. of Chemistry, University of Minnesota, Minneapolis, Minnesota, 55455

[e]Dept of Biochemistry, Molecular Biology and Biophysics, University of Minnesota, Minneapolis, MN 55455

[f] Photonic Pharma LLC, Minneapolis, MN 55410

[g]Dept. of Chemistry, University of Pennsylvania, Philadelphia PA 19104

[h]Biochemistry & Molecular Biophysics Graduate Group, University of Pennsylvania, Philadelphia PA 19104

* Jonathan N. Sachs, corresponding author.

Email: jnsachs@umn.edu

Keywords

Alpha-Synuclein
Toxic oligomers
Soluble oligomers
Small-molecule inhibitors
Time-resolved FRET.

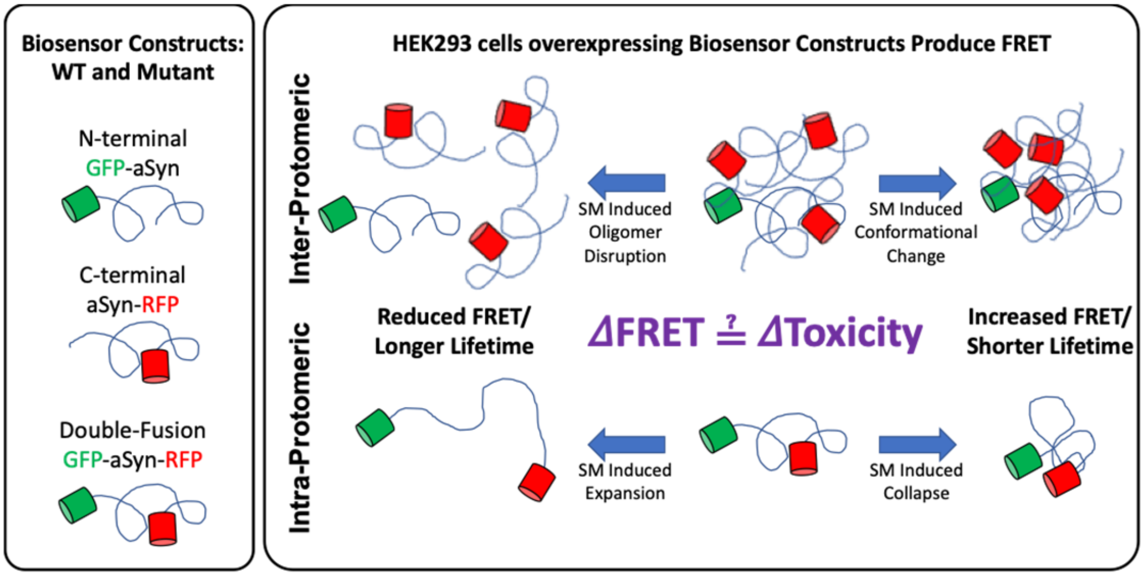

Supplemental Figure 1. Monitoring spontaneous αSN oligomers and conformations via live cell fluorescence lifetime readouts. Inter-protomer (oligomer) and intra-protomeric cellular αSN biosensors use donor and acceptor labeled αSN constructs that produce FRET when overexpressed in cell, indicative of close protein-protein interactions. These two distinct biosensor configurations allow us to monitoring small-molecule induced changes in FRET resulting from: **1)** changes in the extent/amount of oligomerization; **2)** changes in the conformation of monomers within an oligomer; **3)** changes in monomer conformation; or **4)** artifactual interference between the compound and XFP. The inter-protomeric biosensor (top) produced FRET only when oligomers are present. Conversely, the intra-protomeric biosensor (bottom) captures both oligomer induced FRET as well as monomer-conformation driven FRET contributions. These changes in FRET are first confirmed in counter screen systems to rule out potential false-positives and are then correlated to effects on toxicity or direct target engagement through secondary functional assays. In turn, linking the structure and function of modulation of αSN oligomers.

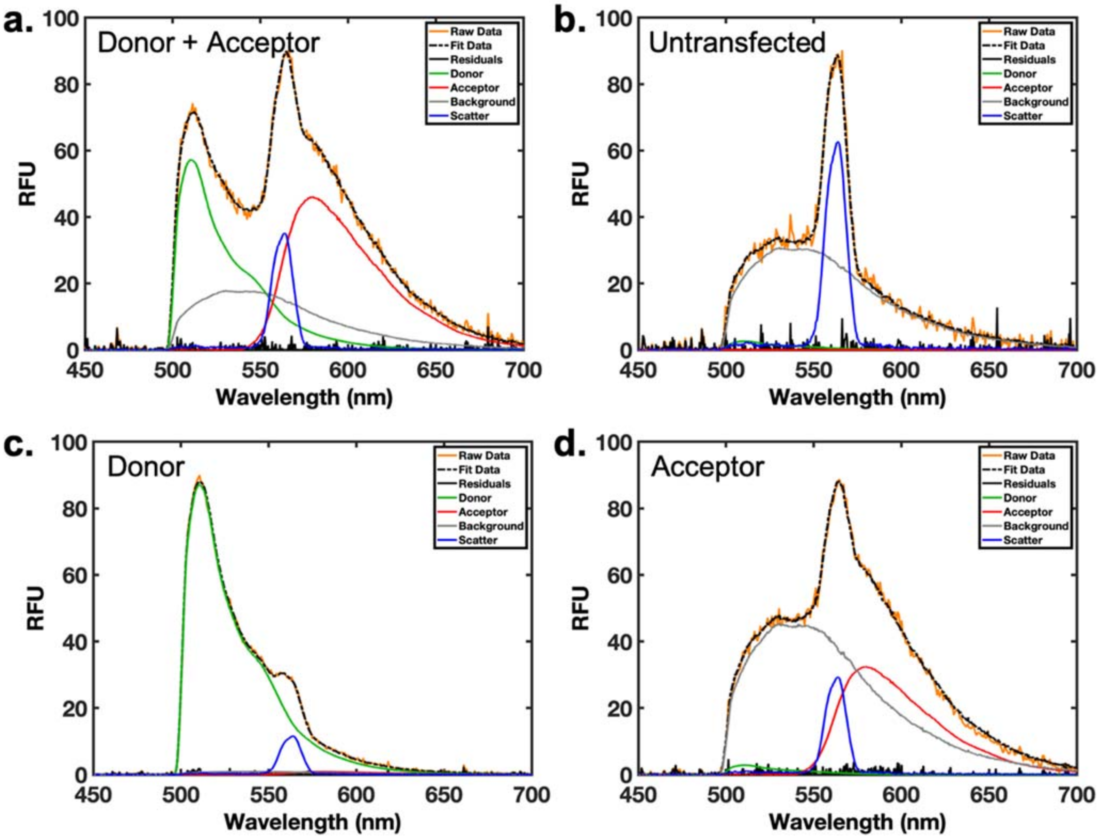

Supplemental Figure 2. Spectral unmixing (using SUPR) decomposes the biosensor's spectra component spectra and provide insight into relative mature XFP. (a) Spectra from the inter-protomeric (oligomer-only) αSN cellular biosensor can be fit to a linear combination of four basis functions: donor; acceptor; cellular autofluorescence background; and Raman scattering (scatter). **(b-d)** Untransfected, GFP-αSN (donor), and αSN-RFP (acceptor) spectra demonstrate spectral unmixing fits for each component. Spectral fitting and interpretation was implemented as previously described[1].

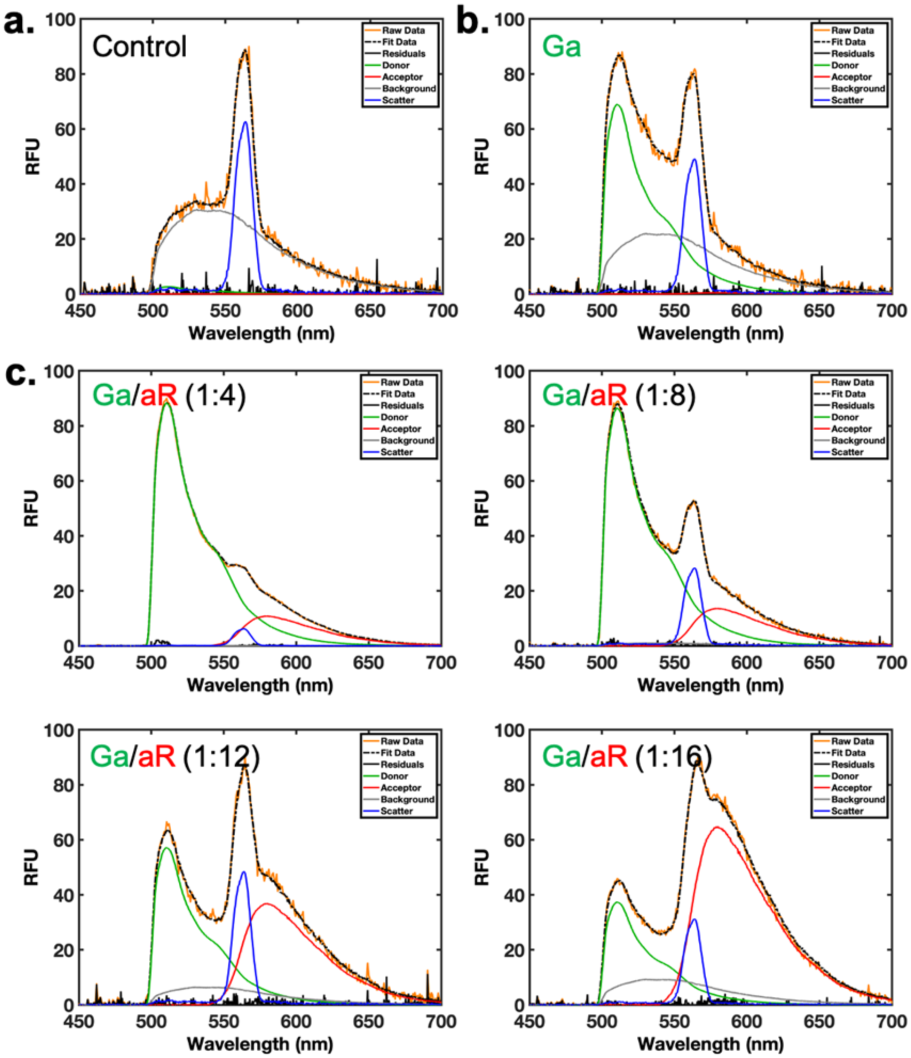

Supplemental Figure 3. Spectra recorded with excitation at 473nm for inter-protomeric αSN biosensor titration (see **Figure 1c** for quantification). With decreasing ratio of transfected donor, the relative donor-to-acceptor contribution becomes more heavily weighted toward acceptor. Concomitantly, as donor intensity decreases the background signal becomes an increasing component to the overall fluorescence. This leads to increased variability in the biosensor's signal as signal to noise decreases and is a limiting factor for selecting an optimal D:A ratio for the biosensor system.

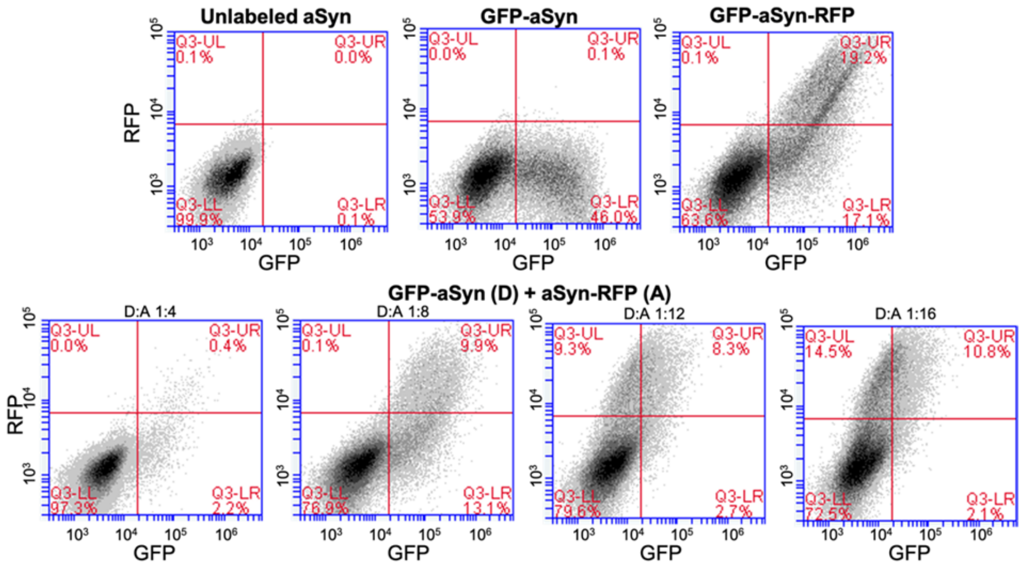

Supplemental Figure 4. Characterization of αSN cellular FRET biosensor expression via Flow Cytometry. Transient transfection of HEK293 cells with the inter-protomeric or intra-protomeric αSN biosensor results in both single- and double-positive fluorescent cells. Optimization of biosensor screening includes evaluation of consistency of double-positive expression.

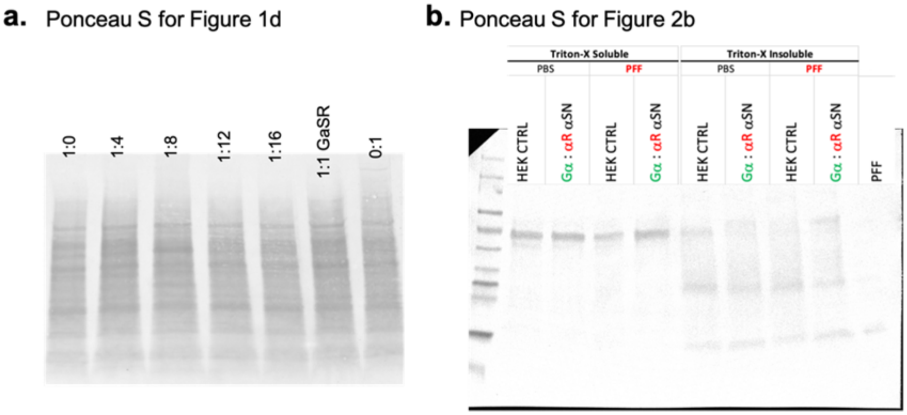

Supplemental Figure 5. Ponceau S loading controls for **(a)** Figure 1d and **(b)** Figure 2b. Differences in the lane profiles for these two different experiments are due to different lysis conditions used in harvesting.

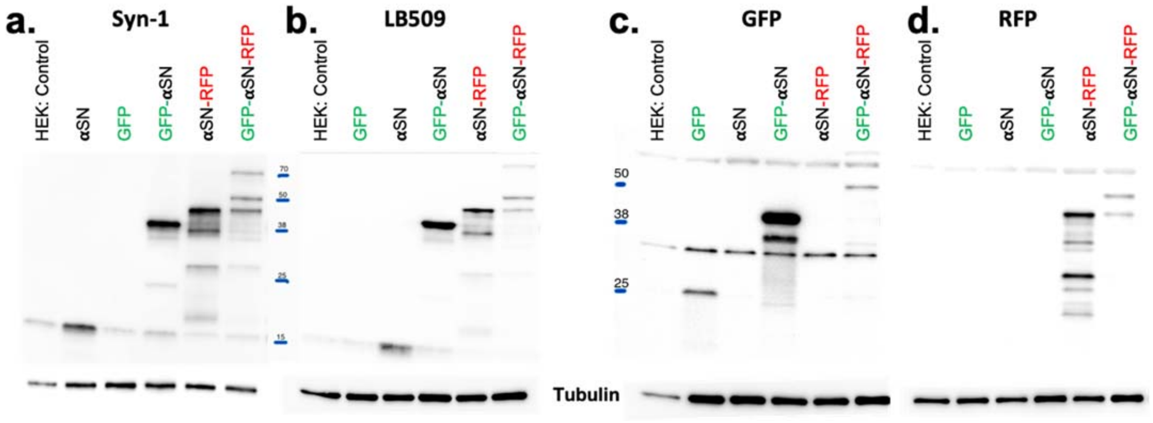

Supplemental Figure 6. αSN cellular biosensor constructs are expressed and truncated. Comparison of Western blots of HEK293 cells expressing different biosensor constructs show the presence of C-terminally truncated αSN with α-Tubulin loading control (bottom). **(a)** The antibody Syn-1 detects a αSN epitope between residues 91-99[2] whereas **(b)** antibody LB509 detects the more C-terminal epitope of αSN (residues 115-122)[3]. In each of the αSN fusion lanes there are bands in Syn-1 that are not present in LB509, indicating a C-terminal truncation of αSN. By looking at the two blots probing for **(c)** GFP and **(d)** RFP we can decipher which fragments of αSN are truncated.

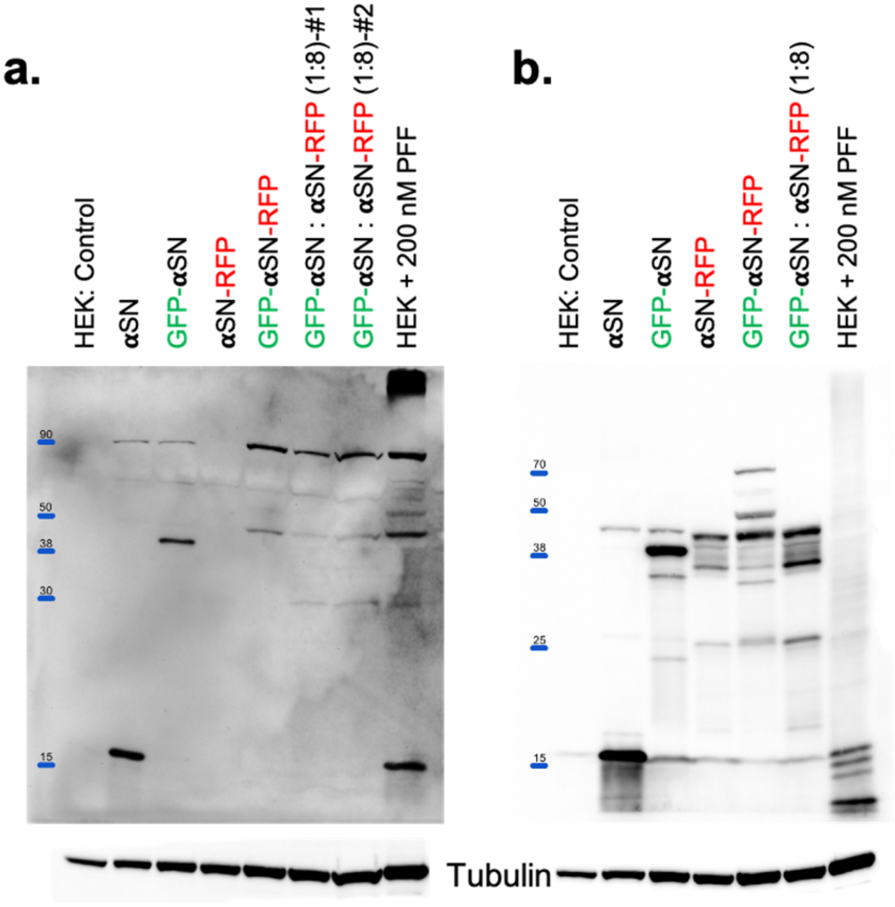

Supplemental Figure 7. Phosphorylation state of αSN cellular biosensor constructs. Western blots of HEK293 cells expressing different biosensor constructs were probed with pS^{129} antibody (**a**) and Syn1 (**b**) demonstrate the phospho-state of Serine-129 when our primary HTS is performed. Unlabeled αSN is expressed much higher than the fusion constructs and has a strong pS^{129} signal. Similarly, GFP-αSN alone displays robust pS^{129}. Other biosensor constructs have some pS^{129} signal but are mostly not-phosphorylated. 200nM PFF treated HEK293 cells were used as positive control.

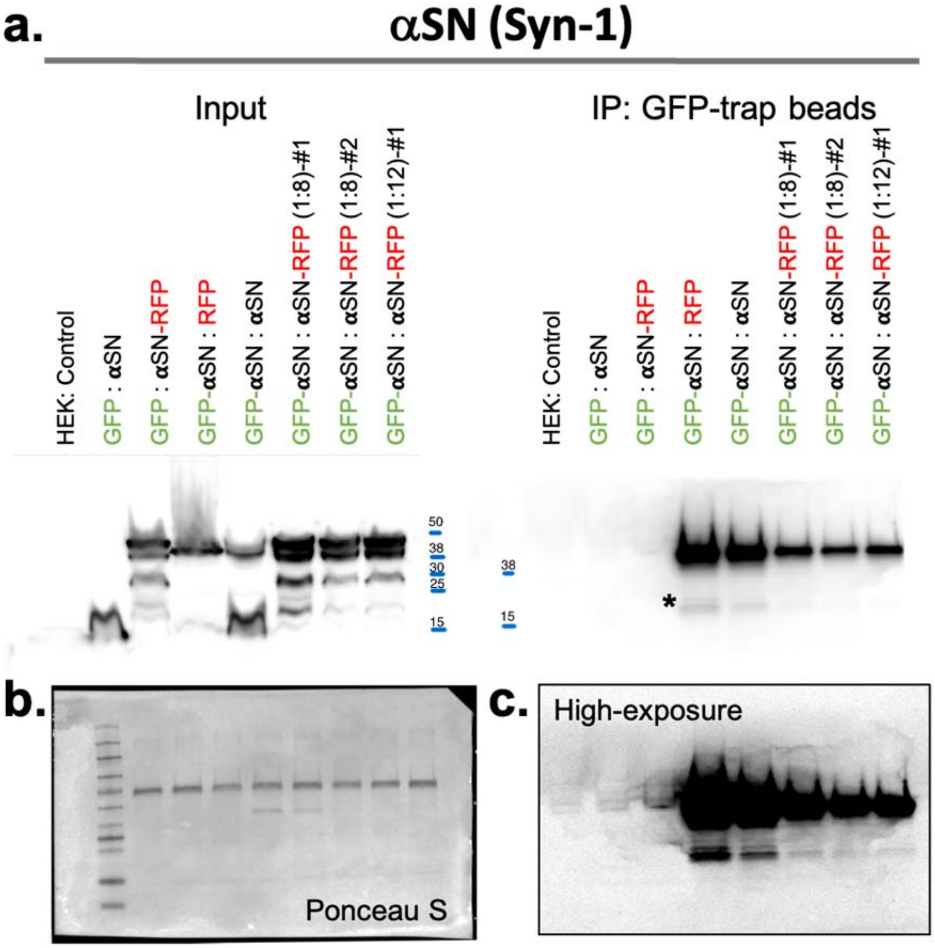

Supplemental Figure 8. Immunoprecipitation of αSN cellular biosensor constructs pulls down endogenous αSN. HEK293 cells were transfected with a series of XFP-fusion constructs, lysed, and immunoprecipitated using GFP-Trap beads. **(a,** *left***)** Western blot analysis of input cell lysates shows a typical banding pattern for αSN biosensor expression with numerous bands indicative of αSN-fusion expression and an array of truncations (see **Supplemental Figure 6**). **(a,** *right***)** GFP-trap beads were used to pull down soluble GFP or GFP-fusion proteins. Only the cell lysates with GFP-αSN were capable of capturing unlabeled, endogenous αSN. In contrast, IP of soluble GFP did not pull down unlabeled αSN. This suggests that αSN/αSN interactions and not GFP/αSN interactions are driving our oligomerization **(b)** Ponceau S loading control for IP samples. **(c)** A long exposure image of **(a,** *right***)** more clearly captures the consistent pull-down of unlabeled αSN by GFP-αSN, whereas soluble GFP only lysate is not capable of capturing unlabeled αSN.

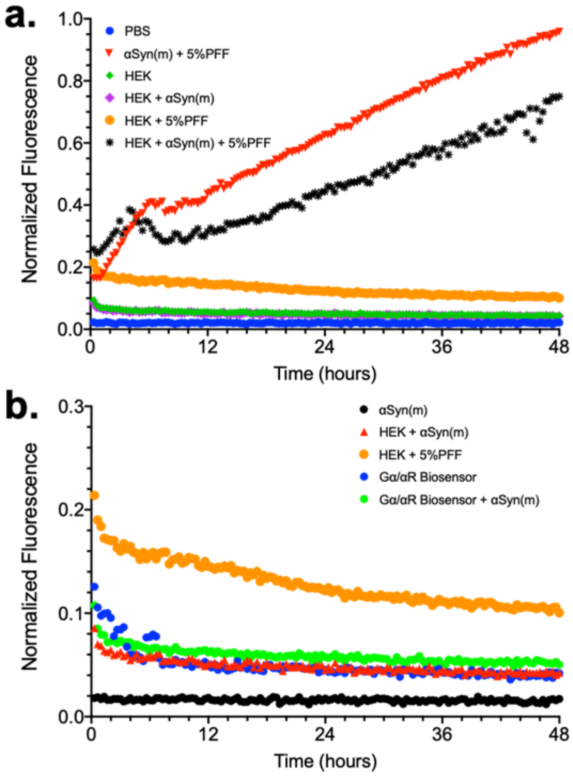

Supplemental Figure 9. The αSN cellular FRET biosensors do not seed fibrils. Inter-protomer (oligomeric) biosensors produce a robust FRET signal from protein-protein interactions. These protein assemblies do not induce seeded fibrillization as monitored via seeded Thioflavin-T (ThT) aggregation assay. **(a)** Control experiments of PBS, αSN+5%PFF, and untransfected HEK293 cell homogenate were incubated in 384-well plate with 900RPM continuous shaking at 37°C for 48-hours. Only samples with both monomeric αSN, αSN(m) at 15µM, and 5% PFF displayed positive ThT signal. **(b)** We observe no seeding capacity for the inter-protomeric αSN cellular FRET biosensors (blue and green) relative to the HEK + αSN(m) + 5%PFF in panel **a** (black).

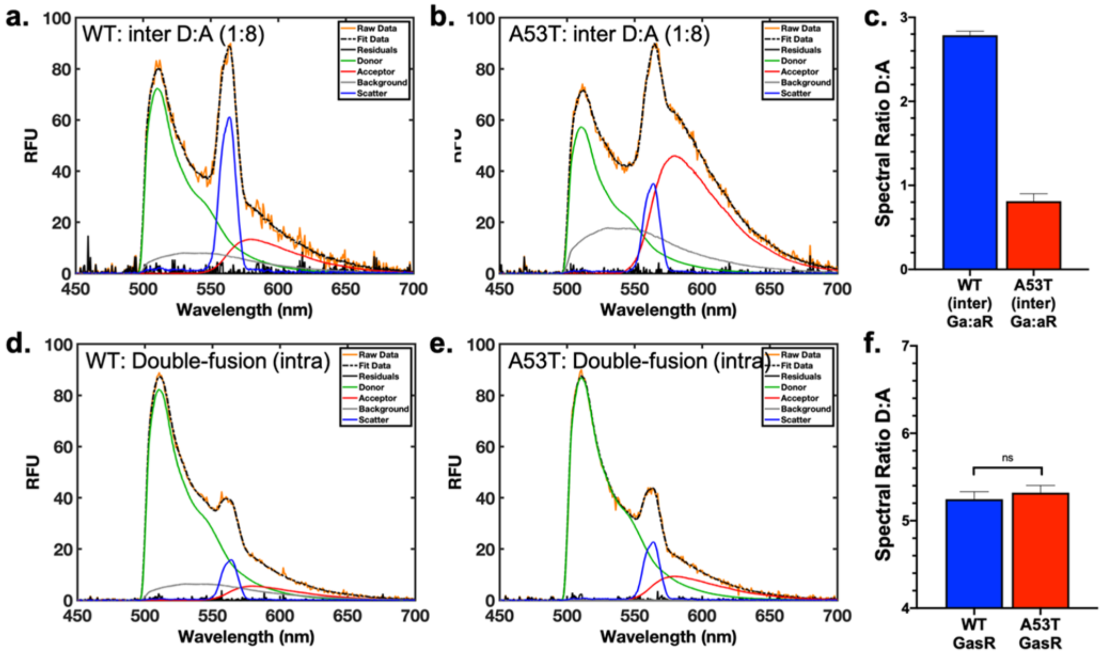

Supplemental Figure 10. Spectral Unmixing of WT and A53T biosensors (a, b) Spectra from inter-protomeric αSN biosensors (WT, **a**; A53T, **b**) illustrates differential donor-to-acceptor expression as quantified in **(c)**. Biosensor FRET signal is sensitive to expression levels which suggest that some of the FRET difference between WT and A53T inter-monomer is potentially due to biosensor expression. However, spectra from the intra-protomeric cellular biosensor **(d, e)** with quantification **(f)** shows consistent expression levels between WT and A53T. Therefore, the observed FRET differences for the intra-protomeric system are due to changes in protein-protein interaction and/or assembly conformation.

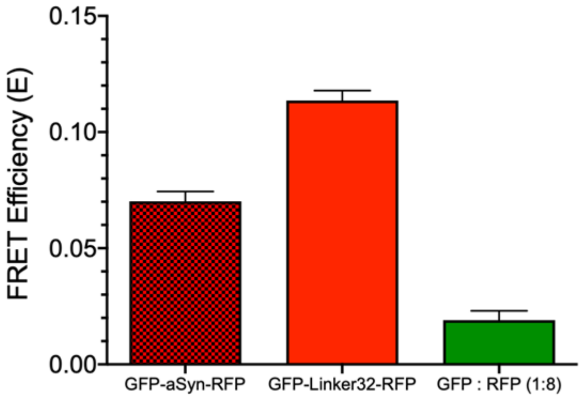

Supplemental Figure 11. Double-fusion intra-protomeric biosensors provide both oligomer and conformation driven FRET signal. Comparisons for the double-fusion (intra-molecular/protomeric) biosensors GFP-αSN-RFP and GFP-linker32-RFP demonstrate the linker-length's potential contribution to overall FRET signal (140 residues in αSN vs. 32 in the linker). Due to the double fusion the D:A ratio is 1:1. Through coupling the double-fusion system with our inter-protomeric biosensor we have better resolution to tease out potential mechanism of action depending on how small-molecules perturb FRET under each condition.

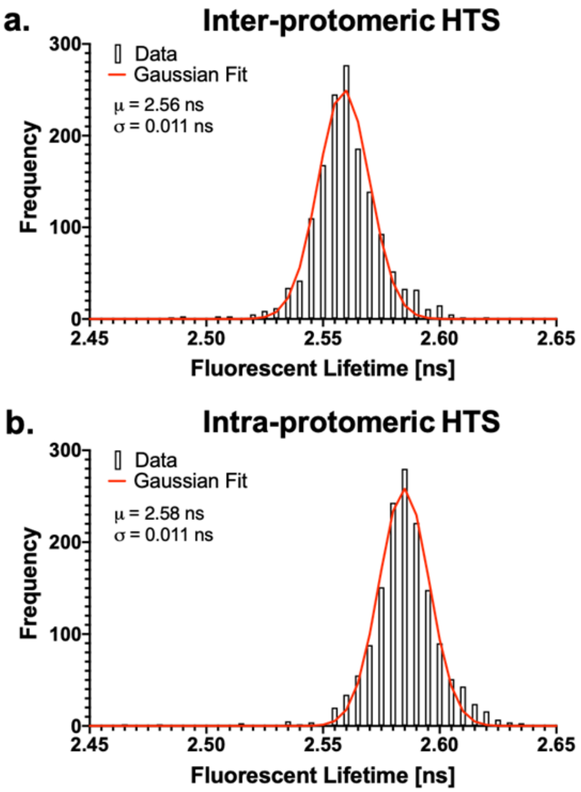

Supplemental Figure 12. Gaussian Fit of Pilot HTS presented in Figure 4. (a) Inter-protomer (oligomeric FRET HTS) and **(b)** Double-fusion (intra-protomeric) pilot-HTS both conform to good agreement with a gaussian FLT response. Mean and standard deviations were used to determine probable hit compounds that are prioritized for subsequent dose-response FRET experiments.

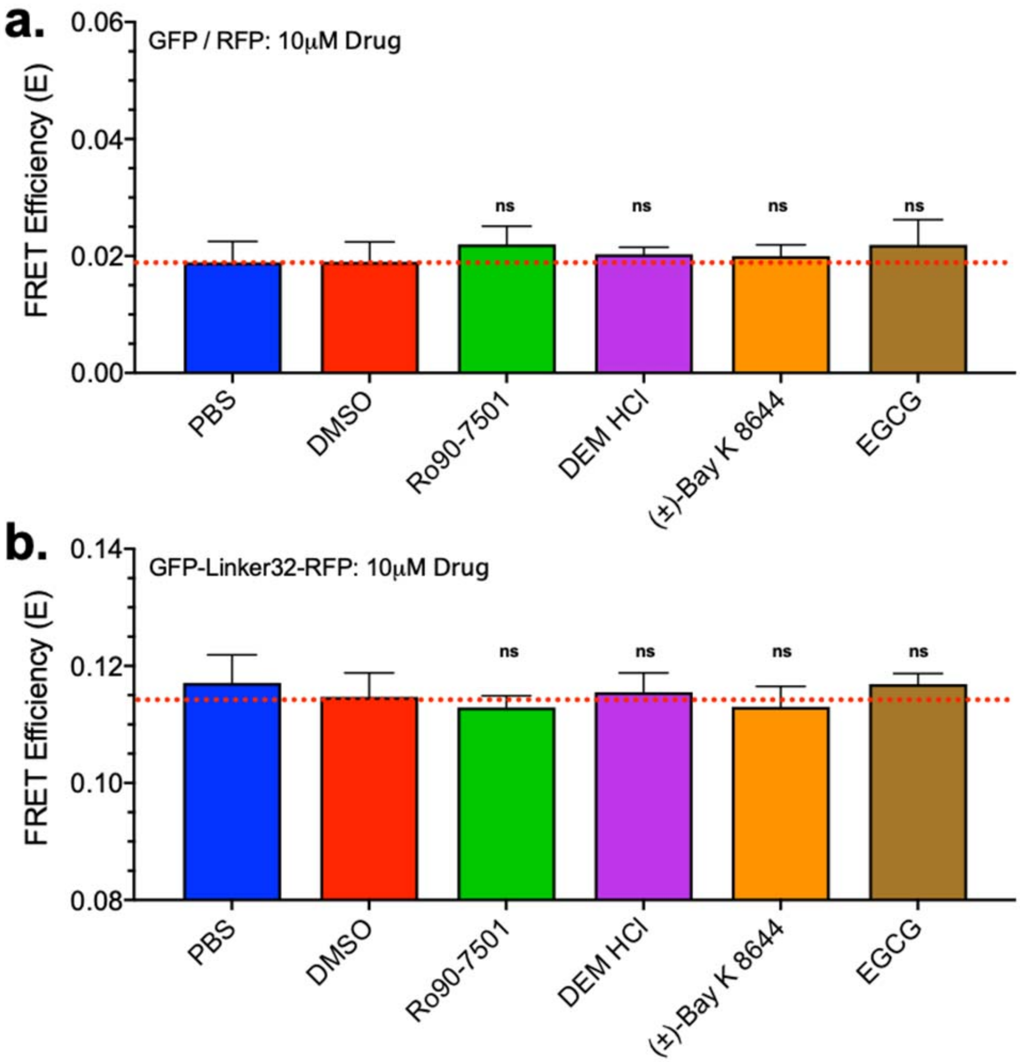

Supplemental Figure 13. Non-specific FRET effects of hit compounds evaluated with control GFP/RFP and GFP-linker-RFP biosensors. Small molecules can induce effects on FRET based biosensors through potential interaction with the XFPs or interference with fluorescence. Evaluation of inter-protomeric non-specific and intra-protomeric effects were performed using **(a)** HEK293 cells expressing soluble GFP/RFP at similar 1:8 DNA titration or with a **(b)** GFP-linker32-RFP double fusion. None of the hit compounds evaluated in this study resulted in a significant change in FRET as determined using student t-test.

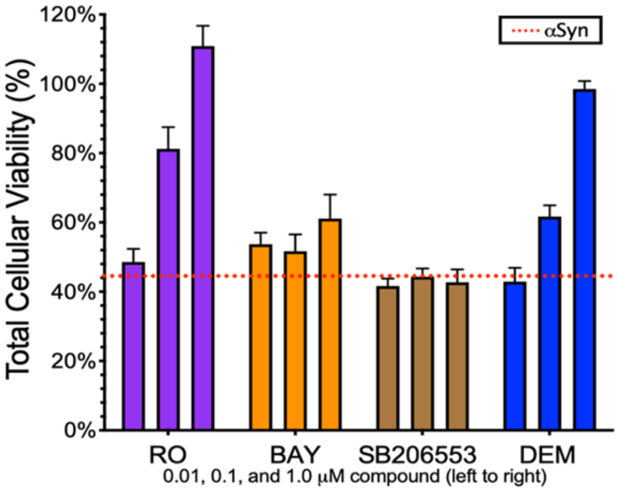

Supplemental Figure 14. Rescue of αSN-induced cytotoxicity in SH-SY5Y cells. Overexpression of αSN in SH-SY5Y cells results in reduced cellular viability (~44%, red dashed line) as determined by CytoTox-Glo cytotoxicity assay. A three-dose assay was used as a preliminary screen for hit-compounds. Not all FRET hits resulted in rescue of toxicity (e.g., RO and DEM show strong response whereas BAY has only mild recovery and SB206553 show no rescue of toxicity.

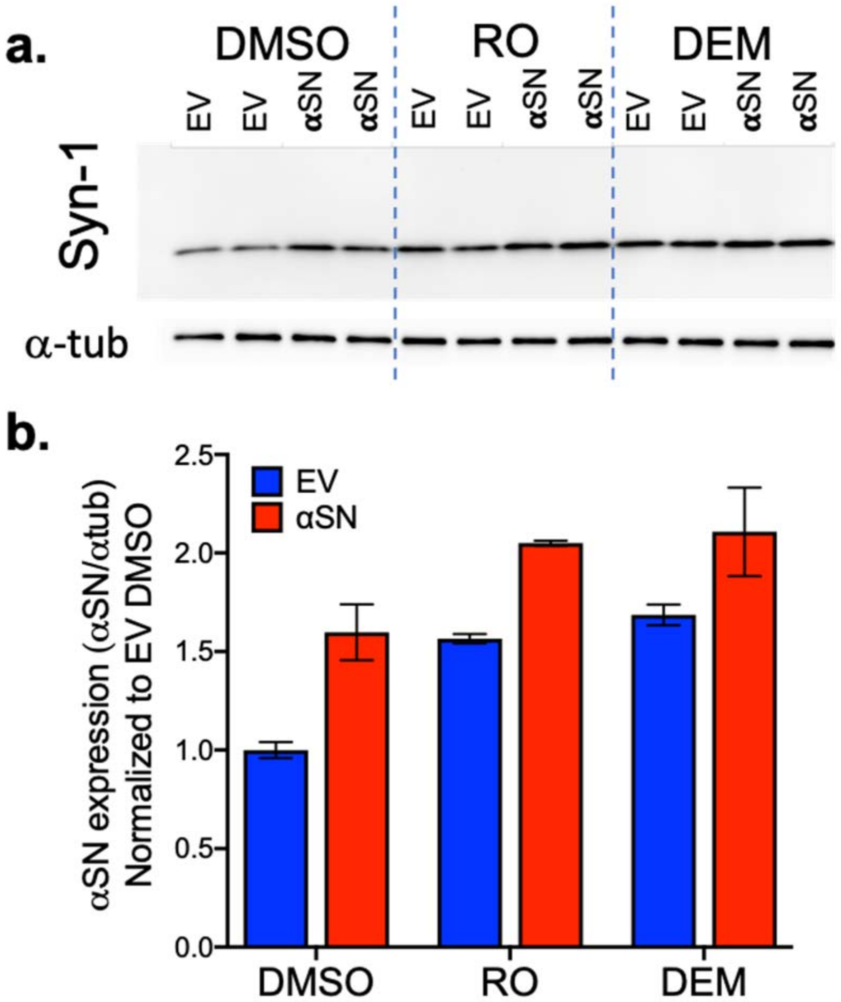

Supplemental Figure 15. Rescue of αSN-induced cytotoxicity in SH-SY5Y cells is not driven by suppression of αSN expression. One potential MOA for our hit compounds could be reduction of αSN levels, thereby alleviating the αSN induced cytotoxicity. **(a)** Treatment of SH-SY5Y cells expressing either αSN or empty-vector (EV) with 1μM compound for 72-hours showed no change in αSN overexpression relative to DMSO for both RO and DEM. **(b)** Quantification of **a** via densitometry analysis.

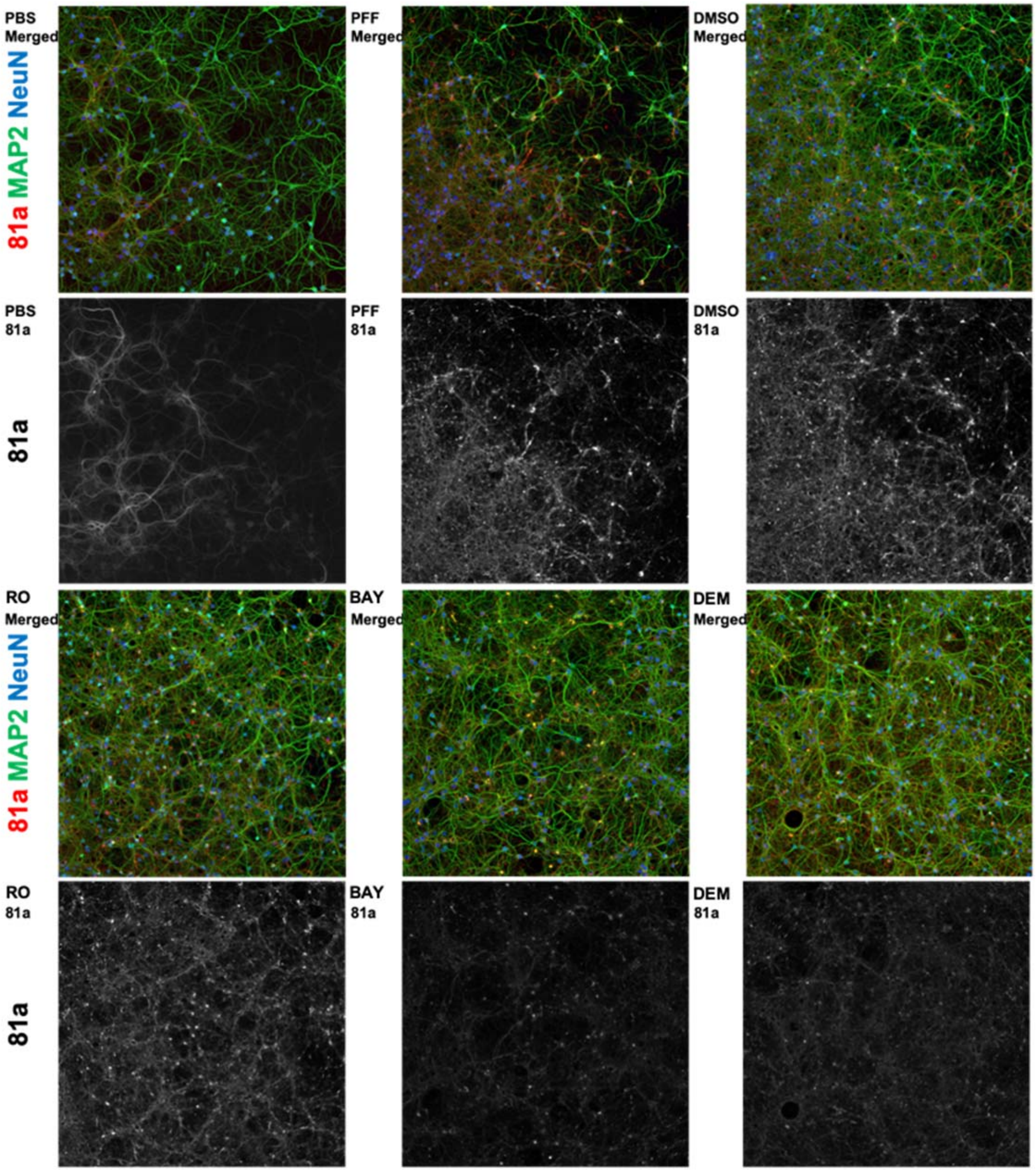

Supplemental Figure 16. Rescue of αSN PFF induced pathology in a primary neuron model. Using a msPFF αSN induced pathology model in primary cortical mouse neurons we monitored phospho-S129 αSN (81a) normalized by the neuron count (NeuN) as a readout for pathology. Neurons were treated with vehicle (PBS), PFF, PFF+DMSO, and three hit compounds (RO, BAY, DEM at 1uM) showed significant increase in 81a/NeuN signal whereas two hit compounds (BAY and DEM) reduced the overall pathology load. Experiments were done in triplicate with three independent wells per experiment. Neurons were cultured for 7DIV, transfected with msPFF +/- compound, incubated 12DIV then processed for IFC. Quantification of NeuN signal is reported in **Supplemental Figure 17** and quantification of 81a/NeuN signal is reported in **Figure 8b.**

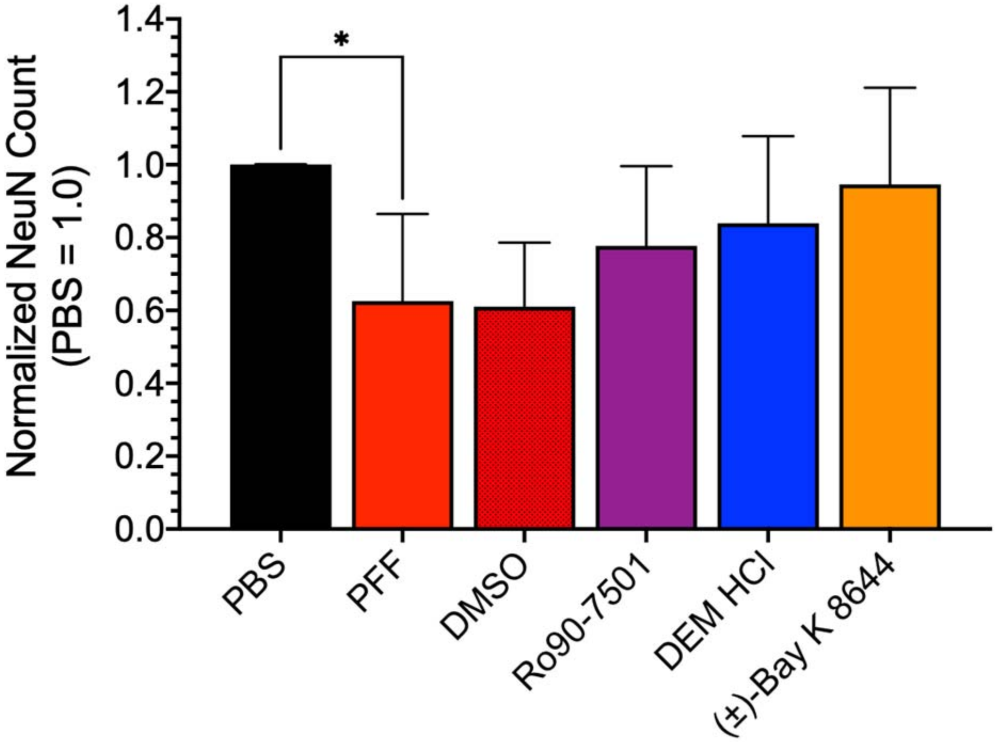

Supplemental Figure 17. PFF induced cytotoxicity in primary mouse neurons. Initial quantification was done on NeuN counts to determine PFF induced cytotoxicity and rescue by our hit compounds. PFFs were treated at ~415ng. NeuN counts for three independent experiments were normalized to PBS control wells. PFFs do induce significant cell death (loss of NeuN signal). Hit compound treatment show a trend to rescue vs. DMSO control. There was significant variability in NeuN counts across experiment and the multiple fields of view used to characterize each condition, likely leading to the trend not being significant.

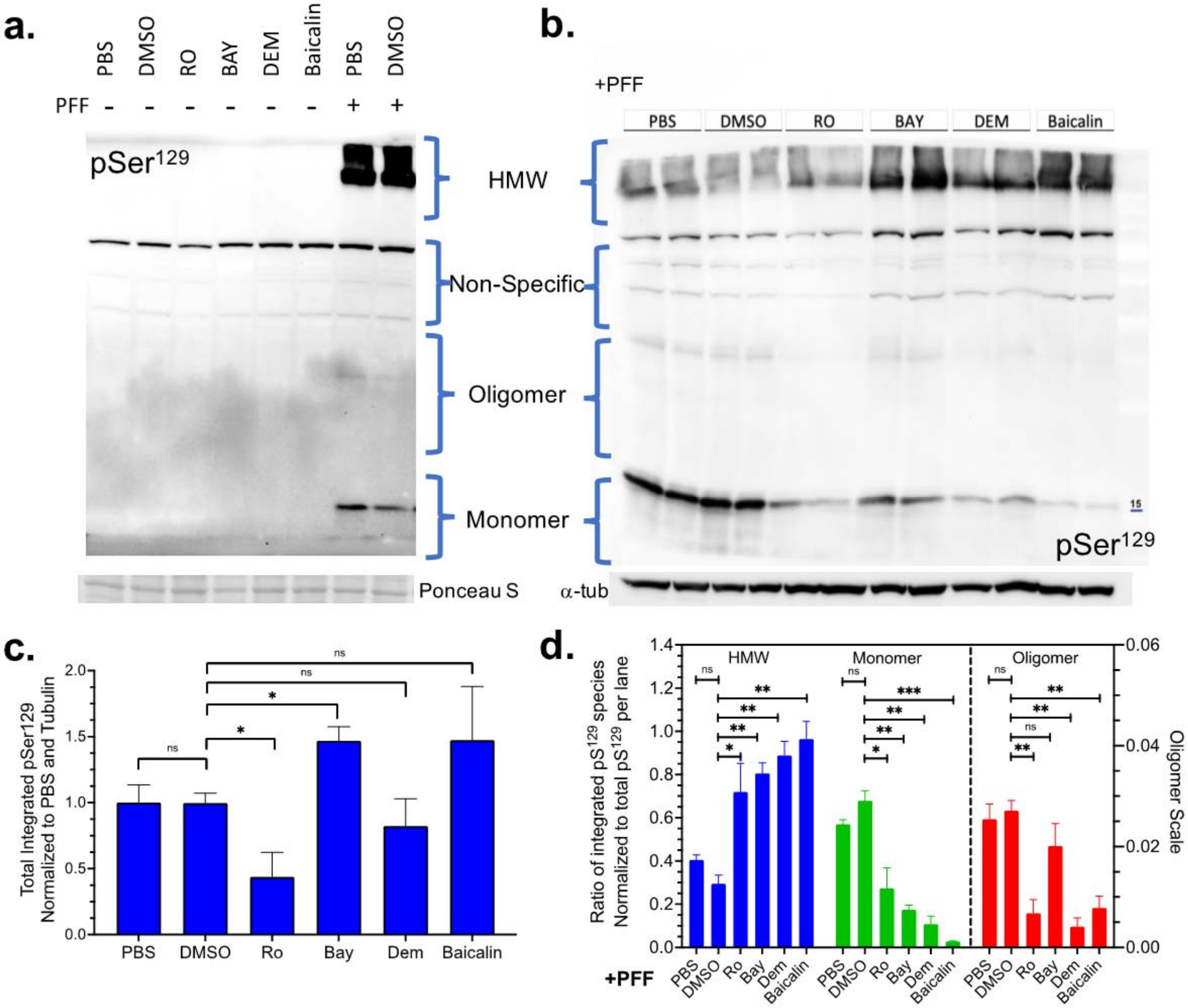

Supplemental Figure 18. Differentiated SH-SY5Y cell model of PFF induced pathology. Differentiated SH-SY5Y cells were treated with PBS, vehicle (DMSO) or compounds with and without 200nM sonicated human αSN PFFs. **(a)** Drug alone does not induce phosphorylation of endogenous αSN however PFF treatment results in clear pS^{129} signal. Note there are multiple non-specific bands present in the untreated samples that persist across samples. These will be removed in subsequent densitometry analysis. **(b)** Replicates of PFF +/- compound treatment were ran for immunoblot analysis of pS^{129} immunoreactivity. **(c)** Densitometry analysis was performed using ImageLab from BioRad. Total pS^{129} signal (excluding the non-specific bands) was normalized to tubulin loading control. Treatment with RO resulted in a significant reduction of total pS^{129} relative to DMSO control whereas Bay resulted in an increased pS^{129} signal. **(d)** Separating the pS^{129} signal into monomer, oligomer, and high-molecular weight (HMW) assemblies highlights a compound induced change in the relative pS^{129} signal for each αSN assembly. Quantification is normalized per lane to highlight change in the ratio of different species. Note, oligomers quantification is plotted relative to the *right-y-axis*. All statistical comparisons were to DMSO treatment using two-tailed t-test with * $p<0.05$; ** $p<0.01$; *** $p<0.005$.

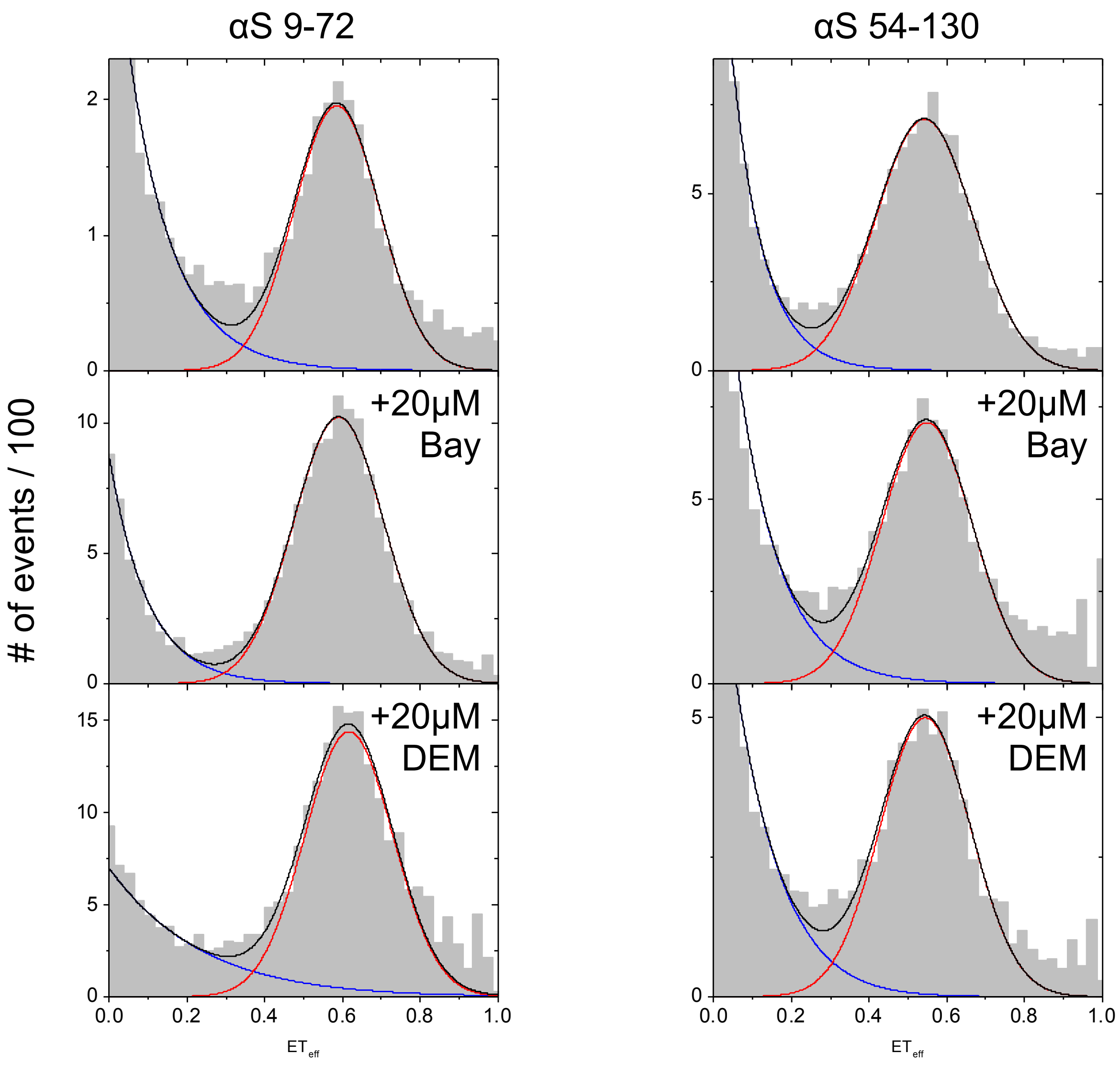

Supplemental Figure 19. Representative histograms from smFRET measurements in the presence and absence of Bay and DEM. αSN labeled at residues 9 and 72 (left) or at residues 54 and 130 (right). FRET measurements of αSN with RO are not shown due to interactions between RO and acceptor dye, Alexa Fluor 594 maleimide.

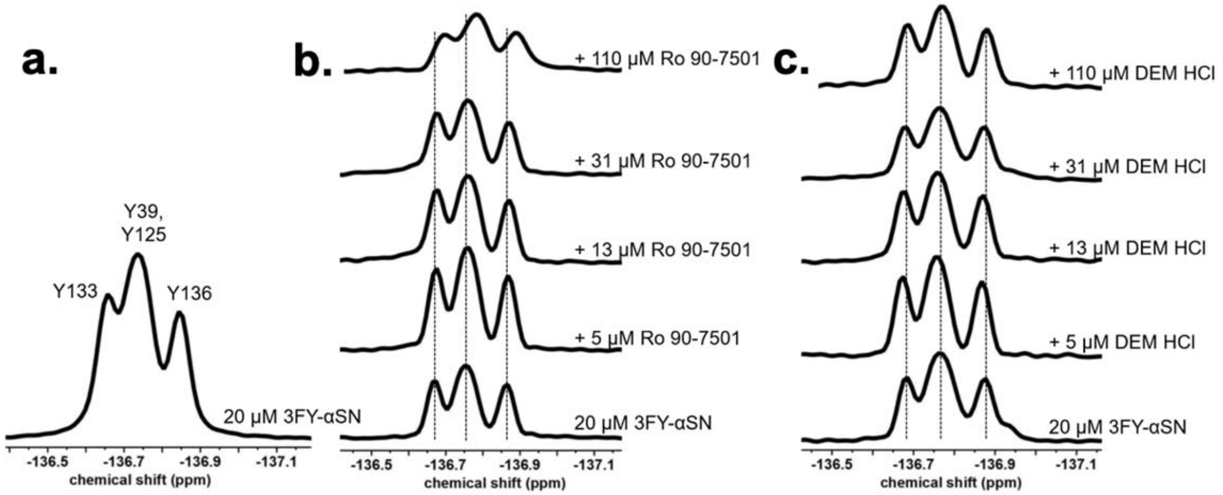

Supplemental Figure 20. PrOF NMR experiments with hit compounds and fluorinated αSN support a lack of binding to monomeric αSN. **(a)** ^{19}F NMR spectrum and assignments of 3FY- αSN. The resulting spectrum is consistent with the spectrum of monomeric 3FY- αSN reported by [4]. Corresponding assignments are used from that report. Stacked ^{19}F NMR spectra with an increasing concentration of **(b)** Ro 90-7501 and **(c)** DEM HCl with 20μM 3FY- αSN. No significant chemical shifts upon compound addition were observed to support compound binding to monomeric αSN. An exception is at high concentrations of Ro 90-7501(110 μM) a small degree of chemical shift perturbation is observed.

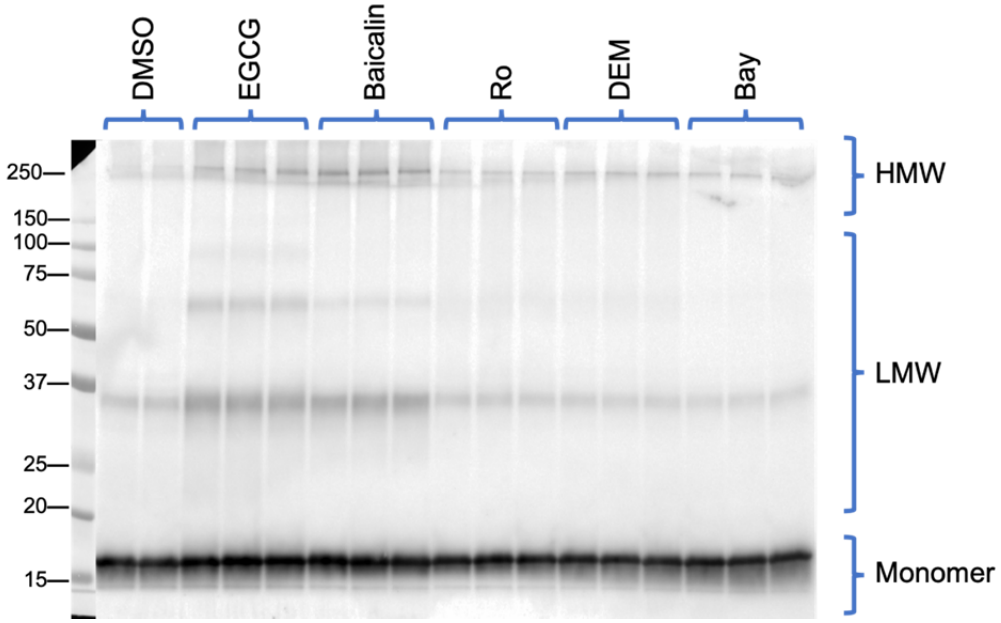

Supplemental Figure 21. Recombinant αSN oligomerization assay quantification gel. Cross-linked aliquots from recombinant αSN oligomerization assays were ran via SDS-page, transferred, and probed for total αSN with antibody Syn-1. Each experiment was performed in triplicate and ran on the same gel for direct comparison. Densitometry quantification for each region (monomer, LMW and HMW oligomers) is presented in **Figure 9a**.

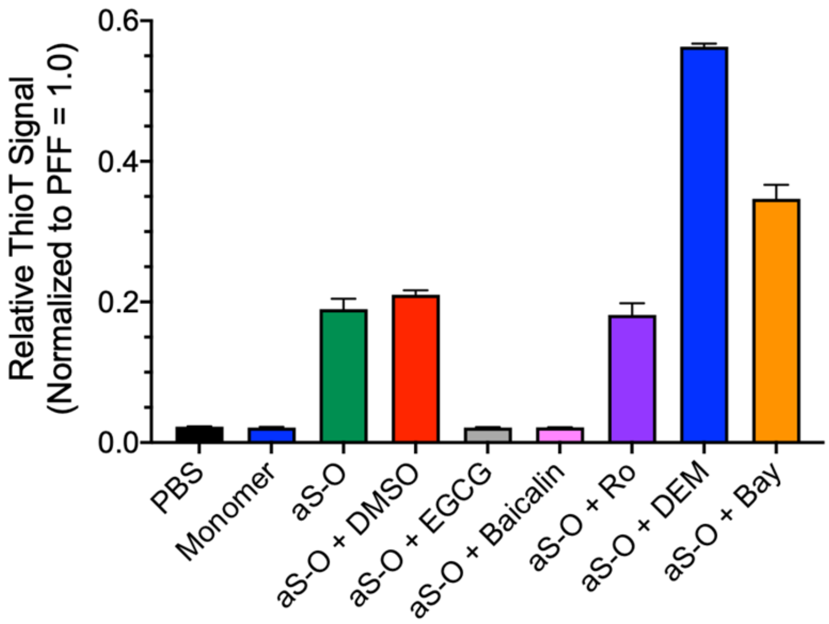

Supplemental Figure 22. Characterization of β-sheet content in αSN oligomers due to hit compound suggests structural remodeling. After the 12-hour oligomerization assay aliquots of each sample were removed and diluted in 100µM ThT and fluorescence read in triplicate (450ex/490em) in 384-well plates. The ThT signal was normalized to a signal from previously frozen αSN PFF aliquots. After completion of the oligomerization assay, the αSN oligomers (aS-O) displayed moderate ThT signal (approximately 20% that of PFF, green bar) and the ThT signal was not affected by DMSO (red bar). Oligomerization in the presence of control compounds, EGCG and Baicalin, at 70µM (a sub-stoichiometric,1:5 compound: monomer ratio) resulted in complete loss of ThT-signal. Our hit compounds RO, DEM, and BAY had differential effects with both DEM and BAY resulting in increased ThT content and RO not producing a significant change relative to DMSO.

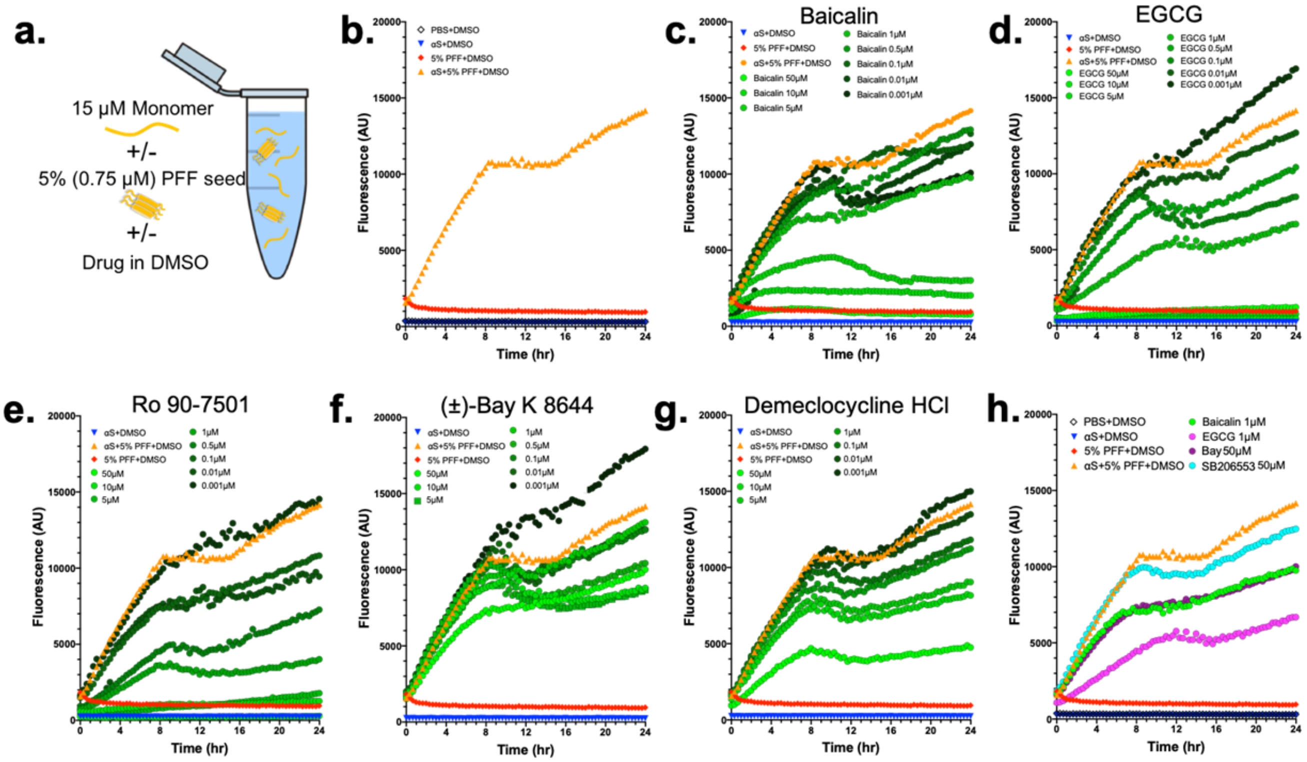

Supplemental Figure 23. Seeded Thioflavin-T aggregation assay show direct protein interaction for subset of hit compounds. (a) Schematic of experiment. Mixtures of 15μM monomer αSN with 5% (0.75μM) PFF were incubated with 20μM ThT and a range of hit compound concentrations. **(b)** Control samples are plotted in all graphs. αSN monomer only (blue), 5% PFF (red), seeded monomer (orange) and PBS only Z(black) provide reference traces. **(c** and **d)** ThT CRC response for control compounds EGCG **(c)** and Baicalin **(d)**. Panels **(e-g)** are ThT CRC for RO, BAY and DEM respectively. Each of these traces were normalized to determine IC_{50} values for inhibition of seeded αSN aggregation (see **Figure 11b**). **(h)** Other compounds tested in ThT assay, SB206553 show no aggregation inhibition. In panels B-H three control samples are included for reference: αSN monomer only (blue); 5% αSN PFF (red), and monomer + seed (yellow).

Supplemental Table 1. Detail list of LOPAC hits for the αSN cellular FRET HTS

	Compound Description			Primary Assay: FRET		Secondary Assay: Functional			Secondary Assay: Biophysical		Previously Known aSyn Compounds
	Compounds	Structure	Known Pharmacological Effect	Biosensor Hit	ΔFLT	1µM Cytotox Response	Cytotox IC50 (nM)	Neuron Response	25µM ThioT Response	ThioT CRC. (µM)	
LOPAC Hit Compounds	Ro 90-7501	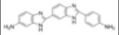	Inhibits amyloid beta42 (Abeta42) fibril formation.	Inter	shorter	+++	78	n.r.	+++	0.931	
	Demeclocycline HCl	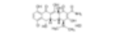	Tetracycline antibiotic; interferes with protein synthesis	Both	shorter	+++	65	++	++	1.00	a*
	(±)-Bay K 8644	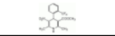	L-type Ca2+ channel agonist	Inter	shorter	+	n.r.	++	n.r.	n.r	
	BIO	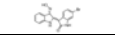	Potent, selective, reversible, and ATP-competitive glycogen synthase kinase 3alpha/beta (GSK-3alpha/beta) inhibitor.	Inter	shorter	+++	150	--	+++**	**	f
	SC-514	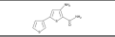	SC-514 is a cell-permeable, potent and selective ATP competitive inhibitor of nuclear factor kappa-B kinase-2 (IKK-2).	Both	shorter	++		n.r. (nc)	n.r.		
	SB 200646 hydrochloride	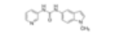	5-HT 2C/2B serotonin receptor antagonist.	Inter	longer	+		-- (nc)	n.r.		
	Retinoic acid	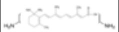	Induces caspase-dependent apoptosis	Inter	shorter	+			n.r.		
	AGK2	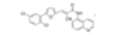	SIRT2 inhibitor. AGK2 rescues dopamine neurons from a-synuclein toxicity in Parkinson's disease models.	Both	shorter	++			++	***	e
	5,5-Dimethyl-1-pyrroline-N-oxide	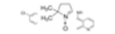	Neuroprotective agent	Inter	shorter	++			n.r.		
	S-Methylisothiourea hemisulfate	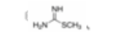	Selective inducle nitric oxide synthase inhibitor	Inter	longer	+			n.r.		
	Gossypol	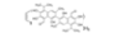	Natural product from cotton seeds with a variety of cell biological activities. Proapoptotic, antimalarial, PKC inhibition.	Inter	shorter	n.r.					
	MRS 2159	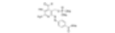	P2X1 receptor antagonist	Both	shorter	--					
	SB 206553 hydrochloride	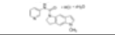	Potent 5-HT2C/5-HT2B serotonin receptor antagonist	Both	longer	++			n.r.		
	Hydrochlorothiazide	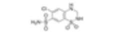	Carbonic anhydrase inhibitor; diuretic	Inter	longer	n.r.					
	Diamide	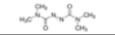	Thiol-oxidizing agent	Inter	shorter	++			n.r.		
	Metergoline	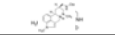	5-HT1/5-HT2 Serotonin receptor antagonist; analgesic; antipyretic	Both	longer	n.r.					
	1-Phenyl-3-(2-thiazolyl)-2-thiourea	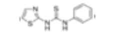	Dopamine beta-hydroxylase inhibitor	Intra	shorter	n.r.					
	PF-4778574	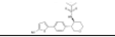	Potent AMPA receptor positive allosteric modulator (PAM).	Intra	longer	+					
	CID2858522	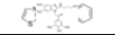	Specifically inhibits NF-kB activation downstream of protein kinsase C (PKC).	Inter	longer	++					
	PD169316	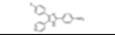	Potent, cell permeable and selective p38 MAP kinase inhibitor; nM potency	Inter	shorter	n.r.					a
	PD173952	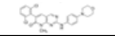	Src family kinase inhibitor.	Inter	shorter	n.r.					a
Control Compounds	Control: Anle138b	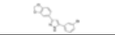	Inhibitor of aSyn and prion protein aggregation and prevents Parkinson's disease in animal models.	Both	shorter	++	900		n.r.		b
	Control: Baicalin	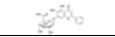	Flavonoid that is an anti-oxidative and anti-inflammtory compound that remodels aSyn fibrils into non-toxic aggregates	Both	shorter	++		n.r.	+++	0.37	c
	Control: EGCG		Polyphenolic compound found in green tea, has anti-oxidative, anti-tumor promoting activity and inhibits aSyn aggregation	Both	shorter				+++	1.10	d

n.r. = no response; nc = negative control; +, ++, +++ = positive response; -- = negative response; ** = artifact due to strong fluorescence; *** = artifact due to drug solubility

a = (41); b = (40); c = (77); d = (32); e = (62); f = (63);

Labeling Positions	E_{eff} (buffer)	E_{eff} (+Bay)	E_{eff} (+DEM)
9C-72C	0.54 ± 0.009	0.54 ± 0.013	0.55 ± 0.005
54-130C	0.59 ± 0.013	0.58 ± 0.005	0.60 ± 0.008

Supplemental Table 2. E_{eff} values for αSN labeled at residues 9 and 72 or at residues 54 and 130 in the presence and absence of Bay and DEM. Values tabulated are mean ± standard deviation for a minimum of three measurements.

**Quikchange #1: Monomerizing EGFP via A206K mutant**	
Forward Primer:	GTCTACCCAATCAAAATTGAGTAAAGATCCGAACGAGAAACGAGATCACATGG
Reverse Primer:	CCATGTGATCTCGTTTCTCGTTCGGATCTTTACTCAATTTTGATTGGGTAGAC
**Quikchange #2: Removing linker and stop codon for αSN**	
Forward Primer:	CTTTAATCAACTCCTCACCTTTGGAAACGGCTTCAGGTTCGTAGTCTTG
Reverse Primer:	CAAGACTACGAACCTGAAGCCGTTTCCAAAGGTGAGGAGTTGATTAAAG
**Quikchange #3: Deletion of mEGFP to make αSN-TagRFP**	
Forward Primer:	GAACCGTCAGATCCGCTAGCGCCACCCATATGGATGTATTCATGAAAGGAC
Reverse Primer:	GTCCTTTCATGAATACATCCATATGGGTGGCGCTAGCGGATCTGACGGTTC
**Quikchange #4: Generating A53T mutant αSN**	
Forward Primer:	CTTTGGTCTTCTCAGCCACTGTTGTCACACCATGCACCACTCCCTCCTTGGTTTTGG
Reverse Primer:	CCAAAACCAAGGAGGGAGTGGTGCATGGTGTGACAACAGTGGCTGAGAAGACCAAAG

Supplemental Table 3. Forward and Reverse primers for all QuikChange mutagenesis reactions used to produce the biosensor constructs.

References:

1 Schaaf, T. M., Peterson, K. C., Grant, B. D., Thomas, D. D. & Gillispie, G. D. Spectral Unmixing Plate Reader: High-Throughput, High-Precision FRET Assays in Living Cells. *J Biomol Screen*, doi:10.1177/1087057116679637 (2016).

2 Perrin, R. J. *et al.* Epitope mapping and specificity of the anti-α-synuclein monoclonal antibody Syn-1 in mouse brain and cultured cell lines. *Neuroscience Letters* **349**, 133-135, doi:10.1016/s0304-3940(03)00781-x (2003).

3 Jakes, R. *et al.* Epitope mapping of LB509, a monoclonal antibody directed against human α-synuclein. *Neuroscience Letters* **269**, 13-16, doi:10.1016/s0304-3940(99)00411-5 (1999).

4 Li, C. *et al.* 19F NMR studies of alpha-synuclein conformation and fibrillation. *Biochemistry* **48**, 8578-8584, doi:10.1021/bi900872p (2009).
